# A novel connection between the Cell Wall Integrity and the PKA pathways regulates cell wall stress response in yeast

**DOI:** 10.1038/s41598-017-06001-9

**Published:** 2017-07-18

**Authors:** Raúl García, Enrique Bravo, Sonia Diez-Muñiz, Cesar Nombela, Jose M. Rodríguez-Peña, Javier Arroyo

**Affiliations:** Departamento de Microbiología II, Facultad de Farmacia, Universidad Complutense de Madrid, IRYCIS, Madrid, 28040 Spain

## Abstract

Fungal cells trigger adaptive mechanisms to survive in situations that compromise cell wall integrity. We show here that the global transcriptional response elicited by inhibition of the synthesis of β-1,3-glucan by caspofungin, encompasses a set of genes that are dependent on Slt2, the MAPK of the Cell Wall Integrity (CWI) pathway, and a broad group of genes regulated independently of Slt2. Genes negatively regulated by the cyclic AMP/Protein Kinase A (PKA) signaling pathway were overrepresented in the latter group. Moreover, cell wall stress mediated by inhibition of β-1,3-glucan synthesis, but not by other cell wall interfering compounds, negatively regulated PKA signaling as indicated by the nuclear localisation of Msn2, cellular glycogen accumulation, a decrease of intracellular cAMP levels and a severe decrease in both the activation of the small GTPase Ras2 and the phosphorylation of known substrates of PKA. All these effects relied on the plasma membrane-spanning sensor of the CWI pathway Wsc1. In addition, caspofungin induced a reduction in the cytosolic pH, which was dependent on the extracellular region of Wsc1. Therefore, alterations of the β-1,3-glucan network in the fungal cell wall, induce, through Wsc1, the activation of the CWI pathway and parallel inhibition of PKA signaling.

## Introduction

Fungi cause a large number of infections worldwide and the incidence of fungi-related infections, primarily in hosts with impaired immunity, has risen sharply over the last few decades. The most significant fungal infections, accounting for approximately 90% of human mortality cases, are caused by species of *Aspergillus*, *Candida*, *Cryptococcus*, and *Pneumocystis* ^1^. The most common classes of antifungals that are currently in use are polyenes, azoles, and echinocandins^2^. Echinocandins are synthetically modified lipopeptides that specifically target the fungal cell wall, a structure that is essential in fungi but absent in animal cells, and they represent the most recent group of antifungal agents introduced into the clinical practice. Echinocandins include micafungin, anidulafungin, and caspofungin (CAS), of which, CAS was the first agent of this group to be approved for therapeutic use^3^. Echinocandin drugs are non-competitive inhibitors of the β-1,3-glucan synthase (GS), an enzyme that is required for the formation of the essential polymer β-1,3-glucan found in the cell wall of several fungal pathogens^4^. Echinocandin resistance has been increasing in recent years^5^, which is of great concern because echinocandin drugs are the main course of therapy for patients with invasive candidiasis or aspergillosis. The resistance to echinocandins fungal pathogens exhibit has been attributed to two mechanisms: mutations that alter the target of the enzyme, the Fks1 catalytic subunit of the GS; and the activation of the signalling pathways required to maintain the cell wall integrity, which leads to an increase in the synthesis of the cell wall components, particularly chitin, which compensates for the inhibition of β-1,3-glucan synthesis due to treatment with echinocandins^2, 6–8^. These compensatory responses are well conserved in fungi and may be clinically relevant as the potential mechanisms of antifungal tolerance.

The Cell Wall Integrity (CWI) pathway, which is the main route responsible for maintaining cell wall homeostasis, has been extensively studied in the model yeast *Saccharomyces cerevisiae* ^9^. When cell wall integrity is compromised, several cell membrane proteins (Mid2, Wsc1-3, and Mtl1) act as sensors of the CWI pathway, which interacts with the guanine nucleotide exchange factor (GEF) Rom2, activating the small GTPase Rho1. This, in turn, activates the yeast protein kinase C (Pkc1). Pkc1 transmits the signal to the CWI MAPK module composed by the MAPKKK Bck1, the redundant MAPKKs Mkk1 and Mkk2, and the MAPK Slt2/Mpk1. Ultimately, Slt2 activates damage-specific transcriptional responses mainly through the Rlm1 transcription factor^9, 10^. In response to being exposed to CAS, yeast cells orchestrate a transcriptional response^11–13^. Moreover, reinforcing the relevance of compensatory mechanisms to tolerate CAS-induced stress, genome-wide screens using *S*. *cerevisiae* have revealed that the synthesis of other cell wall components, such as chitin and mannoproteins, become essential in the presence of CAS^14–16^. It is now recognized that the interconnection of diverse signalling pathways is important to achieving optimum stress responses. In fact, genome-wide surveys of the genes regulated by the CWI pathway are consistent with general stress signalling^17–19^.

Glucose depletion is a type of cellular stress in yeast that, together with other forms of environmental stress, converges on the Msn2 and Msn4 transcription factors^20, 21^. In budding yeast, most of the glucose-induced signalling proceeds through the cAMP/PKA pathway (see refs 22 and 23 for excellent reviews and further details of this pathway). PKA is a heterotetramer that consists of two catalytic subunits that are encoded by *TPK1*, *TPK2*, and *TPK*3, and two identical regulatory subunits that are encoded by *BCY1*. In the absence of glucose, Bcy1 subunits bind to two Tpk subunits to form a catalytically inactive complex. In the presence of glucose, adenylate cyclase (AC) is activated and produces cAMP from ATP. In turn, cAMP binds with Bcy1 releasing it from Tpks and activating PKA. The cellular levels of cAMP are determined by two opposite reactions: Synthesis via AC activity and degradation to AMP by phosphodiesterases encoded by *PDE1* and *PDE2*. AC activity is stimulated by the small GTPases Ras1 and Ras2. The activity of Ras proteins depends on GDP/GTP exchange, where only the GTP-bound form activates AC^24^. A mechanism by which glucose activates the Ras proteins has recently been proposed. Cytosolic pH acts as a cellular signal that activates Ras through the vacuolar ATPase (V-ATPase) in response to glucose availability^25, 26^. PKA influences a wide variety of targets in yeast cells. Globally, it positively regulates the cellular functions associated with fermentative growth and mass accumulation and negatively regulates respirative growth, use of alternative carbon sources, stationary phase or stress response. Remarkably, most of the transcriptional changes that result from the addition of glucose to cells grown in glycerol can be achieved by activating this pathway^27^. An intermediate component in the PKA-mediated regulation of gene expression is the Rim15 protein kinase, which is a regulator of four major transcription factors: Gis1, Rph1, and the partially redundant Msn2/Msn4. These activate gene expression associated to diauxic shift and stress response^28, 29^.

Although information about the global transcriptional response to the cellular damage caused by CAS in wild-type yeast cells is available, little is known about the regulatory mechanisms involved, apart from the activation of the CWI pathway^11–13^. This paper presents new insights into the signalling pathways involved in the regulation of this stress response. Noticeably, besides activating the CWI pathway, CAS also inhibits the cAMP/PKA pathway through the modulation of the intracellular cAMP levels, independently of the CWI pathway MAPK cascade. The cell wall damage observed in these conditions is detected by the extracellular region of the sensor Wsc1, which is required for parallel signalling through both pathways. Therefore, this work improves upon current knowledge on the molecular mechanisms involved in the cellular adaptation response to the cell wall damage caused by inhibition of the beta-1,3-glucan synthase.

## Results

### The transcriptional response elicited by caspofungin depends on both CWI and cAMP/PKA signaling pathways

**Figure 1:**
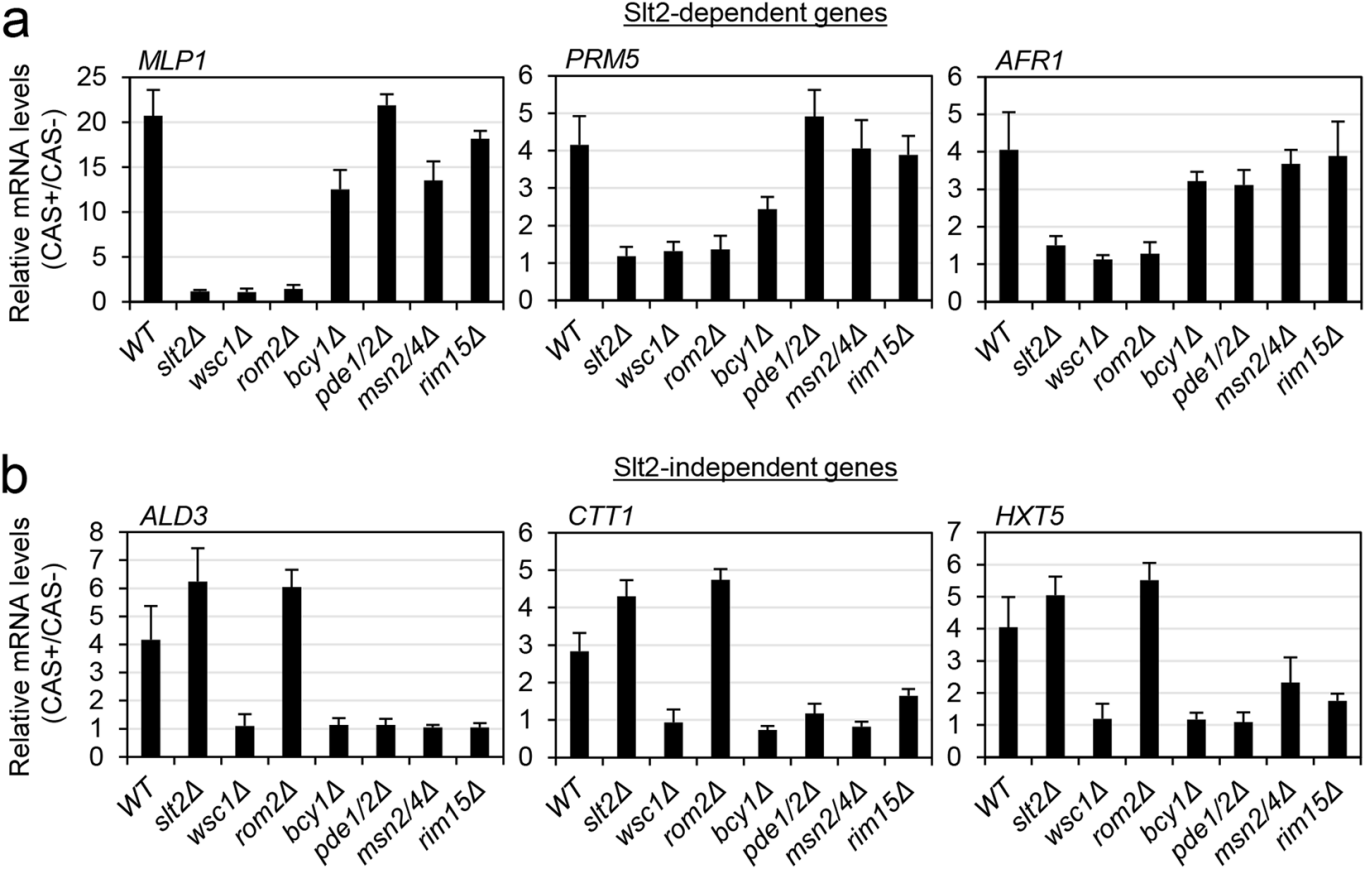
The transcriptional response elicited by caspofungin is regulated by the CWI and cAMP/PKA pathways. mRNA levels of several Slt2-dependent (**a**) and Slt2-independent (**b**) genes were analyzed by RT-qPCR in the indicated strains after 2 h of CAS treatment. Values represent the expression ratio between CAS-treated and non-treated cells. The mean and SD of at least three independent experiments are shown.

CAS treatment activates CWI MAPK Slt2 and this activation is blocked in a strain deleted in the cell surface sensor Wsc1^11, 13^. To further investigate the potential regulatory pathways involved in the transcriptional response to CAS, we obtained, in parallel, the global transcriptional profile of wild-type and *slt2*Δ (MAPK of the CWI pathway) strains grown in the presence and absence of sublethal concentrations of CAS over a period of two hours. In our conditions, a total of 211 genes were up-regulated at least twofold by CAS treatment in the wild-type cells. These data were more similar to those previously published by Agarwal *et al*.^12^ than those published by Reinoso-Martin *et al*.^11^. In fact, although the total number of genes induced in the response described by Agarwal *et al*. was lower, 66% of the genes included in that response was present in ours. In the work by Reinoso-Martin *et al*., CAS only induced the expression of 28 genes. Interestingly, our expression profiles showed that only 72 of the 211 genes up-regulated by CAS treatment in the wild-type cells were not induced in the *slt2*Δ strain (Supplementary Table S1). This was in contrast to data previously reported with Congo red (a chitin-binding dye), where the transcriptional response was almost completely dependent of CWI pathway. We looked for significantly enriched functions (*p*-value < 0.01) using the Gene Ontology (GO) tool “GO Term Finder” within the Slt2-dependent (72 genes) and independent (139 genes) groups. In the first group, as expected, we mainly found genes functionally included in the group of cell wall organization (*p*-value 1.99 × 10^−6^). However, within the Slt2-independent response, enrichment in genes related to small molecule metabolic processes (amino acids, organic acid, and oxidation-reduction metabolism) (*p*-value 9.5 × 10^−4^) and cellular carbohydrate biosynthetic processes (*p*-value 4.2 × 10^−3^) was observed along with a relevant group of genes of unknown function (Supplementary Table S1). As expected, 50% of the genes regulated by Slt2 were induced in other cell-wall stress conditions, such as treatment with Congo red or zymolyase (an enzymatic cocktail including β-1,3-glucanase and protease activities), while the degree of similarity in the Slt2-independent group was very low (Supplementary Table S1). In accordance with the documented role the transcription factor Rlm1 plays in the control of gene expression mediated by Slt2, the *rlm1*Δ strain exhibited a similar behaviour to that exhibited by the *slt2*Δ strain after treatment with CAS (Supplementary Table S1). Thus, those genes that were dependent on Slt2 for induction were also dependent on Rlm1. Since we have previously described how all the transcriptional induction was dependent on the CWI-pathway sensor Wsc1, we employed RT-qPCR to study the effect the deletion of *ROM2*, the principal GEF of the small G-protein Rho1 associated with Wsc1 transduction, had on the induction of representative Slt2-dependent (*MLP1*, *PRM5*, and *AFR1*) and independent (*ALD3*, *CTT1*, and *HXT5*) genes in response to CAS. The results showed that those genes that are dependent on Slt2 and Rlm1 were also dependent on Rom2, whereas upregulation of Slt2-independent genes was not dependent on this GEF (Fig. 1). This indicates that Wsc1 acts via alternative targets to control the branch of the response independently of Slt2.

**Figure 2:**
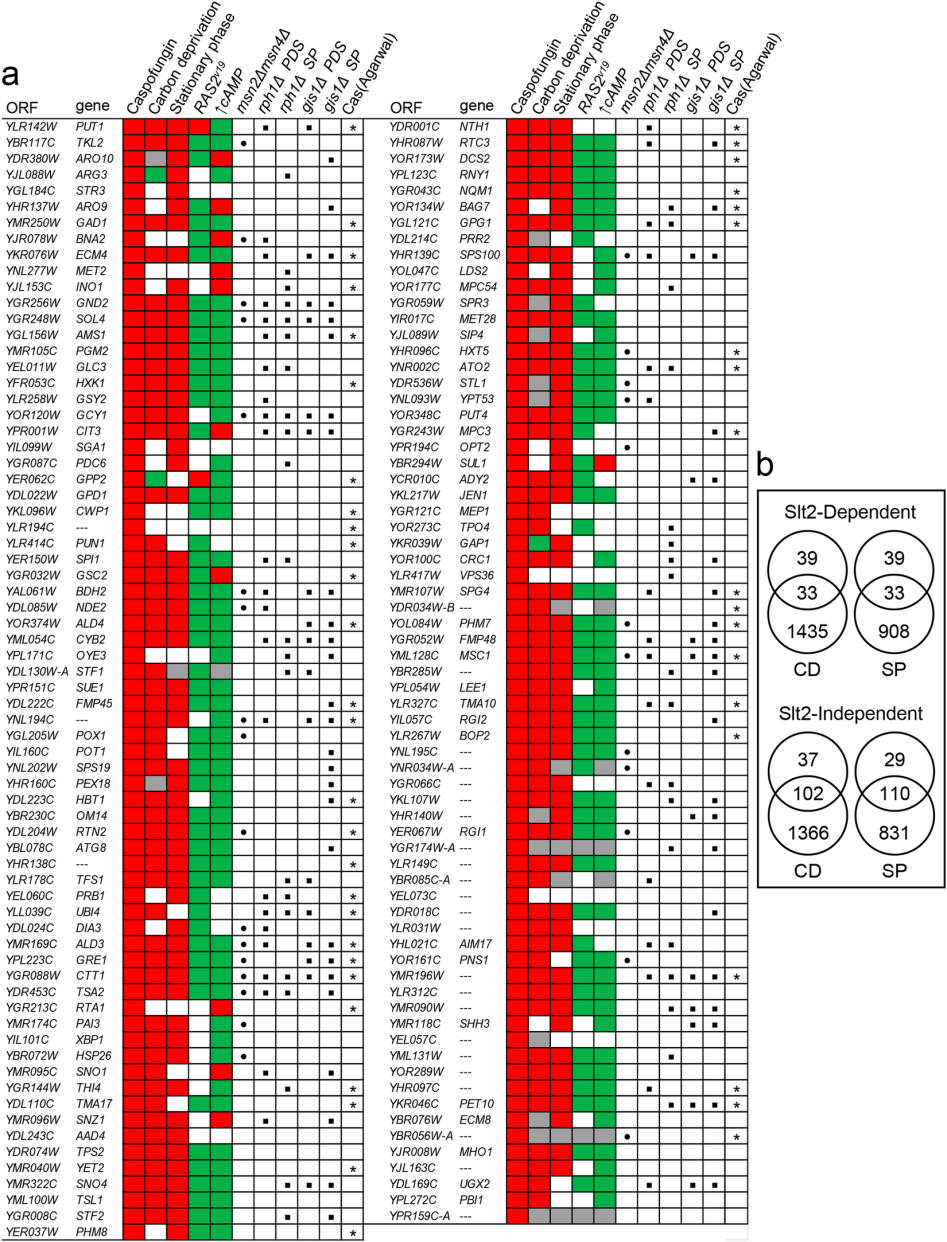
The genes induced by caspofungin independently of Slt2 are targets of the cAMP/PKA pathway. (**a**) Open reading frames whose transcripts were induced at least twofold in experiments of DNA microarrays in the wild-type BY4741 strain, independently of Slt2, after 2 h of CAS treatment, are shown and compared to different transcriptional genome-wide datasets using the MEV (Multiexperiment Viewer) Version 4.9 software from TIGR^67^: Carbon deprivation (YNB lacking D-glucose for 2 h)^68^, stationary phase (YPD medium for 2 days)^20^, *RAS2* ^val19^ (cells expressing a constitutively active allele of *RAS2*) (taken from GEO database GSE8805), and high levels of cAMP (medium including 1 mM cAMP)^69^. Green and red boxes indicate those genes repressed and induced at least twofold respectively in each condition. Gray boxes denote missing values. Those genes that were found to be dependent on Msn2/Msn4 for activation by CAS from DNA microarrays are labeled with a black dot. Black squares indicate those genes previously reported to be dependent on Rph1 and Gis1 transcription factors after diauxic shift (PDS) (wild-type strain grown in glucose depletion for 9 h) and/or stationary phase (SP) (wild-type strain grown in YPD for 3 days)^29^. Those genes previously described upregulated in the study of Agarwal *et al*.^12^ are marked with an asterisk. (**b**) Comparison of the overlap of Slt2-dependent or independent genes with those induced by stationary phase (SP) or carbon deprivation (CD).

The identification of a significant number of genes that were induced in a Slt2-independent manner indicated that other signalling pathways were potentially involved in the regulation of the response to CAS. To study these alternative regulatory circuits, a bioinformatic analysis of the microarrays data using the web-based application MARQ^30^ was performed. This tool facilitates a comparison of a query set of genes from a given experimental condition against a signature database built from GEO datasets (the gene expression data repository of the NCBI) for different organisms and platforms to identify the experimental conditions that induce similar gene expression profiles. As a result, the expression patterns associated with response to nutrients were principally identified (sharing more than a 70% of genes, *p* < 0.0001) within the group of genes induced by CAS independently of Slt2. As shown in Fig. 2a,b, the patterns identified included both gene upregulation, such as conditions of stationary phase or carbon deprivation, or opposite regulation, as in the case of the expression of a dominant active allele of the small GTP-binding protein Ras2 (Ras2^val19^) or the presence of high levels of cAMP. These data suggested that CAS inhibits the cAMP/Protein kinase A network, which plays a critical role in cell growth and response to glucose. We further investigated whether the transcriptional profile triggered by CAS was also conserved in response to other inhibitors of the β-1,3 GS, by genome-wide expression profiling of wild-type yeast cells treated with an alternative echinocandin, aminocandin^31^. This echinocandin induced the expression (ratio +/− drug ≥2) of 300 genes, including 189 out of the 211 genes upregulated by CAS (Supplementary Table S2). Moreover, in agreement with the high level of similarity found in the transcriptional response elicited by both compounds, within the group of 111 genes specifically upregulated under aminocandin treatment, 92 had expression ratios ≥1.5 for CAS (Supplementary Table S2). These results suggest that the complete transcriptional effect induced by CAS, including PKA inhibition, is due to its activity on β-1,3 GS complex.

To further investigate the potential PKA signalling pathway collaboration in the control of the transcriptional response to CAS, we used RT-qPCR to measure the transcriptional induction levels of the Slt2-independent genes *ALD3*, *CTT1*, and *HXT5* in a *bcy1*Δ strain and a double mutant *pde1*Δ*pde2*Δ. These strains were selected because Bcy1 exhibits an inhibitory activity on the PKA catalytic subunits, and the absence of phosphodiesterase activity (*pde1*Δ*pde2*Δ) leads to an increase in intracellular cAMP levels and, thus, also to the activation of PKA. As shown in Fig. 1b, the increase in the expression of these genes in the *pde1*Δ*pde2*Δ and *bcy1*Δ mutants was completely blocked. On the contrary, the expression levels of Slt2-dependent genes (*MLP1*, *PRM5*, and *AFR1*) induced by CAS were similar to those observed in the wild-type strain (Fig. 1a). These results support the notion that the effect on gene expression caused by CAS treatment relies on the CWI and cAMP/PKA pathways.

To predict the potential transcription factors involved in the regulation of the Slt2-independent response, the genes included in this group were analysed for documented regulatory associations based on experimental data using YEASTRACT (www.yeastract.com). The principal regulatory associations are shown in Table 1. As expected, the transcription factor Rlm1 was consistently associated with the group of genes controlled by Slt2, whereas in the case of the genes that are non-regulated by Slt2, the partially redundant transcription factors Msn2/4, globally associated with response to stress, were the most significant. Moreover, 80% of these genes bore in their promoter regions at least one binding element for Msn2/4 (STRE element, AGGGG) (Table 1). To gain further insights into the role of Msn2/4 in the CAS-mediated transcriptional induction, we analysed the global transcriptional profile of a double mutant strain, *msn2/4*Δ, grown in the presence or absence of CAS. The findings revealed that 29 out of the 139 genes upregulated by CAS in the wild-type strain independently of Slt2 were not induced in the *msn2*/*4*Δ strain (Fig. 2 and Supplementary Table S1). Moreover, in agreement with previous findings that PKA inhibits the protein kinase Rim15, which, in turn, functions as a transcriptional activator of Msn2/4 target genes^32^, expression of selected Msn2/4-dependent genes was blocked in a mutant *rim15*Δ grown in the presence of CAS (Fig. 1b). In contrast, the induction of Slt2-dependent genes was not affected by the deletion of *MSN2/4* or *RIM15*. Remarkably, we found STRE and PDS (Post Diauxic Shift, AGGGAT) motifs in 79% and 37% respectively of the Slt2 and Msn2/4 independent genes. Interestingly, it has previously been reported that a relevant number of genes within this set is regulated by the PKA-related transcription factors Gis1 and Rph1 after diauxic shift (glucose depletion) and/or stationary phase (Fig. 2). Gis1 acts primarily on PDS motifs after diauxic shift, while Rph1 acts on genes with STRE motifs^29, 33^. Therefore, all these data are compatible with a scenario in which CAS induces the transcription of a set of genes negatively regulated by PKA in a context in which multiple transcription factors operate in a coordinated manner.

**Table 1:** Transcription factor regulation enrichment for genes included in the genome-wide transcripcional response to caspofungin.

Transcription Factor	% DRA	*p*-value	% TF-binding sites	Description
**Slt2**-**dependent genes**				
Rlm1	81.94	0.00E+00	66.67	CWI MAPK pathway
Gcn4	75.00	3.12E-09	68.05	Amino acid biosynthesis
Ste12	80.56	6.95E-07	25.00	Mating MAPK pathway
Tec1	79.17	1.76E-06	80.55	Filamentous growth
Ace2	87.50	1.82E-05	22.22	Cell separation (M/G1 transition)
**Slt2**-**independent genes**				
Msn2	94.24	0.00E+00	80.57	Response to stress
Msn4	86.33	0.00E+00	80.57	Response to stress
Gcn4	86.61	0.00E+00	61.15	Amino acid biosynthesis
Bas1	92.09	0.00E+00	30.21	Purine and histidine biosynthesis
Swi5	75.54	0.00E+00	25.17	M/G1 phase gene expression
Tec1	79.86	2.47E-11	79.86	Filamentous growth
Ace2	89.21	2.59E-10	25.17	Cell separation (M/G1 transition)
Ste12	75.54	5.02E-08	17.26	Mating MAPK pathway

DRA, percentage of genes showing documented regulatory associations with transcription factors (*p*-value < 0.001) as deduced from analysis with YEASTRACT. Those transcription factors with DRA percentages higher than 75%, ordered by *p*-value, are shown. Percentage of genes showing in their promoters at least one DNA-binding domain for each indicated transcription factor is also included.

### Caspofungin negatively regulates PKA signaling

**Figure 3:**
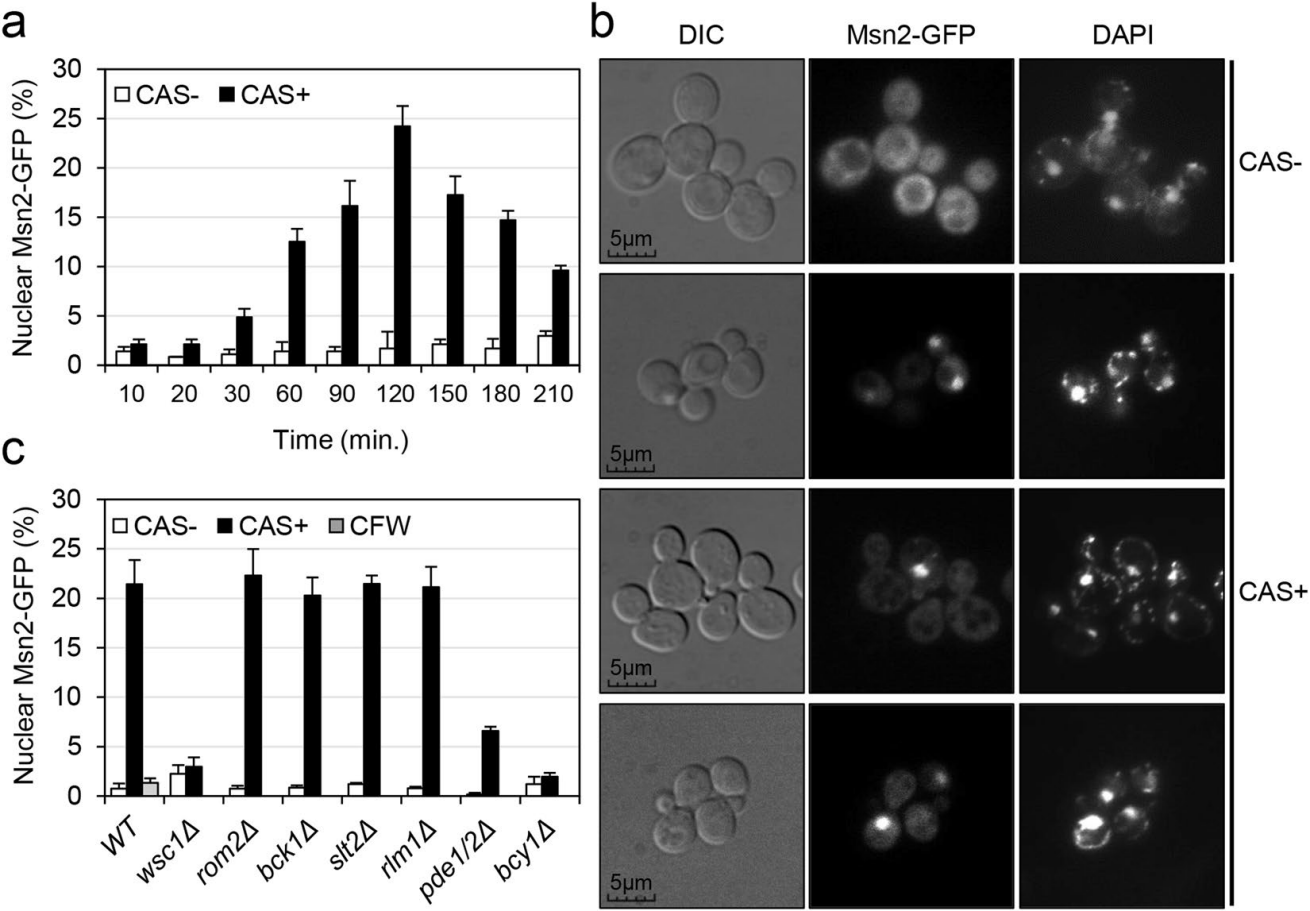
Caspofungin treatment triggers nuclear entry of the Msn2 transcription factor. (**a**) Histogram depicts the percentage of cells showing nuclear Msn2-GFP localisation in the wild-type strain at the indicated times of CAS (15 ng/ml) exposure. (**b**) Exponential cultures of the wild-type BY4741 (WT) strain expressing Msn2-GFP were subjected (CAS+) or not (CAS−) to CAS (15 ng/ml for 2 h), collected, and studied under fluorescence microscopy. Staining with DAPI is also shown. In living cells, DAPI stains mitochondrial and chromosomal DNA. (**c**) Histogram depicts the percentage of cells showing nuclear Msn2-GFP in the wild-type and the indicated mutant strains grown in the presence or absence of CAS over 2 h. Nuclear localisation of Msn2-GFP after Calcofluor white treatment (CFW, 5 µg/ml for 2 h) was also measured for the wild-type strain. Values represent the average and SD calculated from three independent experiments (at least 300 cells were analysed for each independent count).

Having established a connection between the components of the PKA pathway and a branch of the transcriptional response elicited by CAS, we further analysed if signalling through this pathway was affected by the presence of this drug. In this context, intracellular distribution of Msn2 is closely linked to the status of PKA activity. Low PKA activity leads to nuclear accumulation of Msn2, whereas high activity causes nuclear Msn2 redistribution to the cytoplasm^34^. We thus monitored nuclear/cytoplasmic localisation of Msn2 in wild-type cells expressing Msn2-GFP grown in the presence or absence of CAS, in a time-course experiment from 0 to 210 minutes of treatment. The kinetics of Msn2 localisation revealed an evident gradual increase in the percentage of cells with nuclear localisation of Msn2-GFP after 30 minutes of drug exposure reaching a peak at 2 h (Fig. 3a). Nuclear translocation of Msn2-GFP after 2 h of treatment, evidenced by simultaneous GFP and DAPI cell fluorescence visualization, is shown in Fig. 3b. After the 2 h peak, the amplitude of Msn2 nuclear localisation decreased slowly over time (Fig. 3a). Contrary to other types of stress in which the Msn2 nuclear translocation generally occurs quickly (less than 10 minutes) and affects a high percentage of the cell population, CAS induces this phenomenon in a lower percentage of cells and more delayed over the time.

In agreement with transcriptional data, the effect on the Msn2-GFP subcellular pattern caused by CAS in a wild-type strain was completely abrogated in the mutant strains *wsc1*Δ and *bcy1*Δ and drastically reduced in the *pde1*Δ*pde2*Δ strain (Fig. 3c). On the contrary, elements of the CWI pathway, namely, Rom2, Bck1, Slt2, and Rlm1, were not required for the Msn2-GFP nuclear translocation induced by CAS. Remarkably, the effect of CAS appeared to be cell-wall damage specific, since treatment with Calcofluor white, a chitin-binding dye that has a well-documented cell wall damaging activity, did not induce nuclear Msn2-GFP localisation (Fig. 3c).

**Figure 4:**
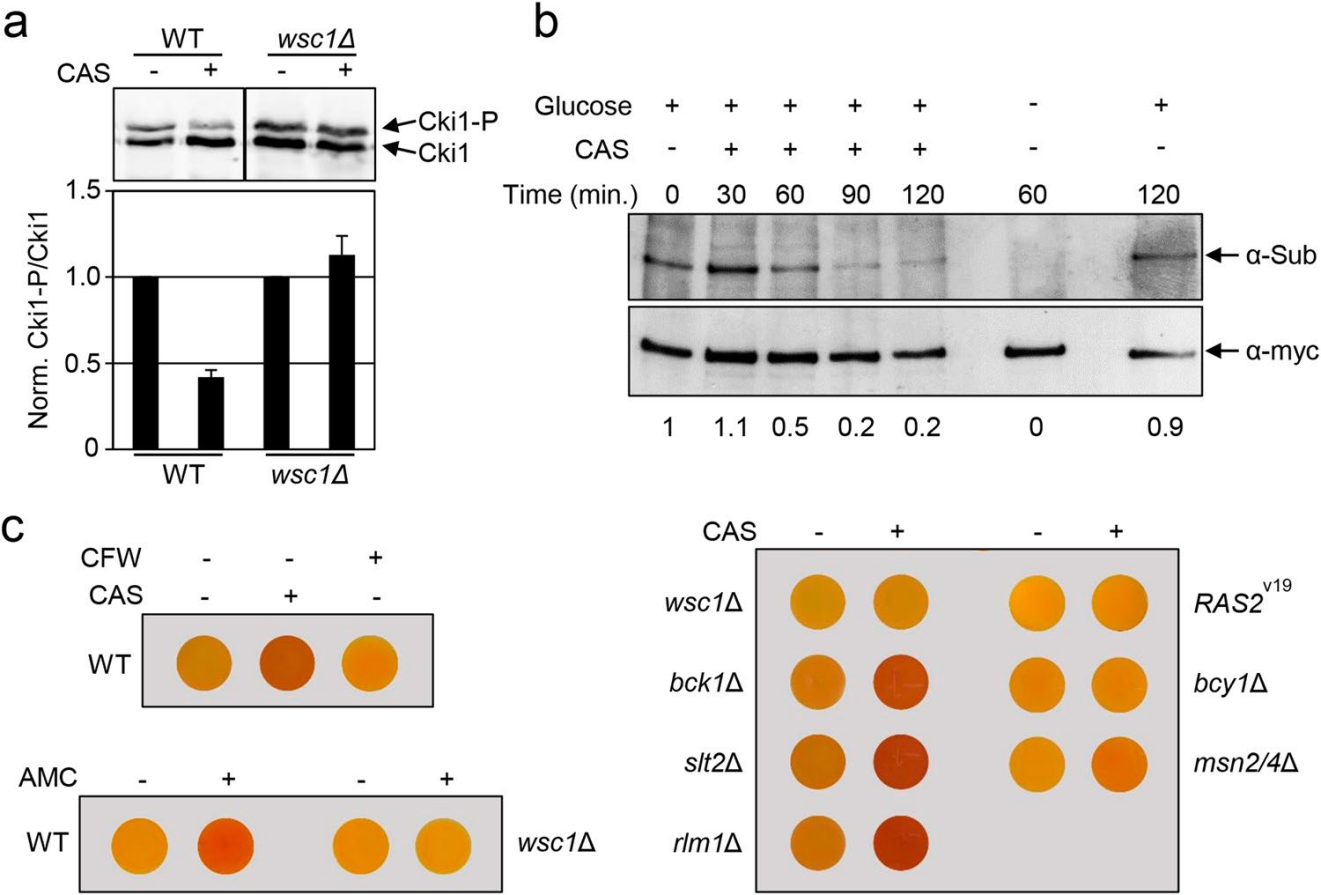
Caspofungin controls PKA activity in a Wsc1-dependent manner. (**a**) Wild-type (WT) and *wsc1*∆ cells harboring pRS423-pr^*CUP*^-6xMYC-*cki1* ^*2_200*(*S125/130A*)^ plasmid were grown in YPD medium in the absence and presence of CAS for 2 h. PKA-dependent phosphorylation of Cki1 was analyzed by whole cell extraction and Western blot analysis using an anti c-myc antibody. A representative Western blot is shown (upper panel). Ratios of phosphorylated (Cki1-P) and non-phosphorylated (Cki1) forms of Cki1 in the presence of CAS relative to untreated cells are shown for each strain in the lower panel. The means and SD of three (n = 3) independent experiments are indicated. (**b**) The PKA-dependent phosphorylation of Pat1 was lost upon CAS treatment. Wild-type cells expressing a myc-tagged Pat1 were grown to mid-log phase in YPD medium and then treated or not with CAS (15 ng/ml) or transferred to a medium lacking glucose (YP) for the indicated time. The level of PKA phosphorylation (α-Sub) was assessed by Western blotting with the anti-PKA substrate antibody. Numbers indicate the relative amounts (quantified by densitometric analysis) of Phospho-Pat1 with respect to time zero normalized with respect to total Pat1 (α-myc). (**c**) Glycogen accumulation is induced in the presence of CAS. Cells of the indicated yeast strains (*RAS2* ^val19^ corresponds to the wild-type strain expressing this *RAS2* allele) were grown on YPD medium in the absence or presence of CAS (15 ng/ml), Calcofluor white (CFW, 5 µg/ml), or aminocandin (AMC, 15 ng/ml) for 2 h. Cells were then collected and resuspended in a solution of 0.2% iodine/0.4% potassium iodide and, after 3 min., were spotted onto YPD plates and photographs were taken. The darker the color, the higher the amount of glycogen that was accumulated. Glycogen provides an indirect measurement of cAMP/PKA activity.

To further demonstrate *in vivo* the inhibitory effect of CAS on PKA activity, we firstly followed the phosphorylation of the PKA substrate Cki1. To achieve this, we used a Cki1 variant that is exclusively phosphorylated by PKA^35^. PKA-dependent phosphorylation of the Cki1 reporter was detected by a mobility shift in SDS-PAGE analysis, and the ratio of phosphorylated and non-phosphorylated forms was quantified as a measure for the *in vivo* PKA activity in cells as previously described^36^. In exponentially growing cells exposed to CAS, we observed a sixty-percent decrease in the Cki1-P/Cki1 ratio compared with that measured in cells grown in the absence of stress (Fig. 4a). Again, in accordance with the transcriptional data, the effect of CAS on the Cki1-P amount was totally dependent on the Wsc1 sensor. We further confirmed this effect on PKA activity by monitoring the *in vivo* phosphorylation levels of another well-characterized PKA substrate, the RNA metabolism-related protein Pat1, using an antibody that specifically recognizes sites that are phosphorylated by PKA^37^. Wild-type cells expressing a myc-tagged Pat1 were grown in the presence of CAS and samples were taken at different times, namely 30, 60, 90, and 120 minutes. Next, myc-Pat1 was immunoprecipitated from whole cell extracts corresponding to each time point, and the levels of PKA phosphorylation were scored. As shown in Fig. 4b, CAS induced a time-dependent dephosphorylation of Pat1, with this effect being evident after 60 min of CAS treatment. After 90–120 min, only a residual PKA phosphorylation of Pat1 was detected. Interestingly, kinetics of PKA-dependent Pat1 dephosphorylation was completely different to that previously associated with glucose deprivation, another well-characterized physiological condition in which Pat1 phosphorylation by PKA is completely lost after only 10 minutes of glucose deprivation^37^.

Additionally, we analysed the accumulation of glycogen as an additional readout of PKA inhibition^38^. We measured glycogen accumulation due to CAS treatment in wild-type cells, CWI pathway mutants, and PKA-related mutants. Whereas wild-type, *bck1*Δ, *slt2*Δ, and *rlm1*Δ strains treated with CAS were stained dark brown by iodine staining, indicative of glycogen accumulation, the *wsc1*Δ mutant and those cells lacking Msn2/4 or with PKA constitutively activated (*bcy*1Δ and *RAS2* ^val19^ expression) exhibited no glycogen accumulation (Fig. 4c). Moreover, treatment with aminocandin also provoked glycogen accumulation in a Wsc1-dependent manner (Fig. 4c), reinforcing the notion that the inhibition of the β-1,3 GS is responsible for the effects observed on the cAMP/PKA pathway. In contrast, a different type of cell wall stress as that caused by Calcofluor white treatment did not induce glycogen accumulation (Fig. 4c), in accordance with the lack of effect of this drug on Msn2 localisation described above. Collectively, all these results demonstrate that *in vivo* activity of PKA is negatively modulated after yeast cells are exposed to CAS.

### Ras2-GTP and cAMP levels decrease in the presence of caspofungin

**Figure 5:**
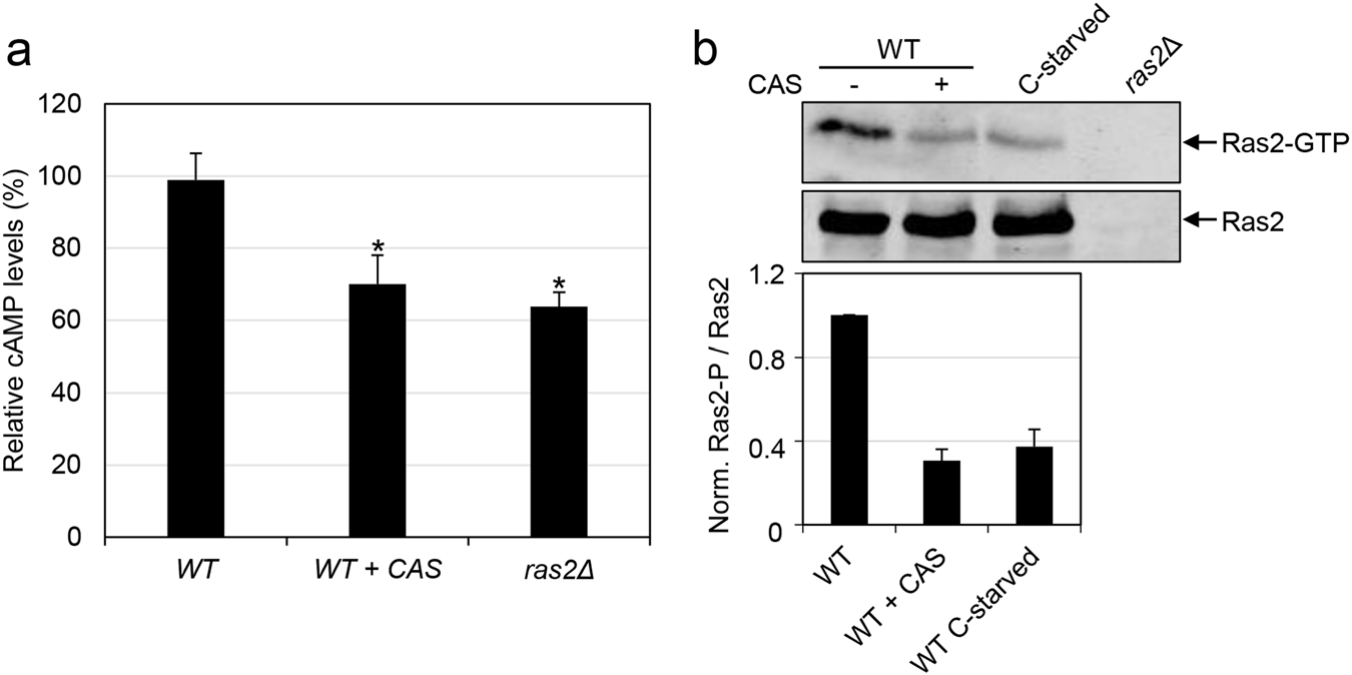
Intracellular cAMP and Ras2-GTP levels decrease in cells treated with caspofungin. (**a**) Wild-type (WT) was grown in YPD medium in the absence or presence of CAS during 2 h for cAMP quantification. The *ras2*Δ mutant was included as control. Relative cAMP levels are shown (wild-type strain = 100%) from three independent experiments. Statistical analysis was carried out using a two-tailed, unpaired, Student’s t-test to analyse differences between the wild-type strain treated with CAS or the *ras2∆* mutant versus the wild-type strain without treatment: 0.01 ≤ *p ≤ 0.02. (**b**) Whole cell extracts were prepared from early log phase cells of the wild-type (WT) strain grown in YPD in the presence or absence of CAS for 2 h, the *ras2*Δ strain grown in YPD and WT cells grown in SC medium transferred to SC-D for 2 h (conditions of glucose starvation, C-starved). Active Ras2 (Ras2-GTP) was pulled down using GST-RBD bound to glutathione beads. The levels of Ras2-GTP (in the pull-down samples) as well as total Ras2 (in the whole cell extracts) were detected by immunoblotting with the anti-Ras2 antibody. A representative Western blot is shown. The mean and SD from three independent experiments of the ratio Ras2-GTP/total Ras2 relative to the wild-type sample (ratio = 1) is presented in the lower panel.

The direct activator of PKA is cAMP, which activates this kinase by binding to its regulatory subunits and relieving their inhibition on the catalytic subunits. We decided to determine the effect of the presence of CAS in the growth medium on cellular cAMP levels. The levels of cAMP were determined during exponential growth in the wild-type strain in the presence or absence of CAS and, in parallel, in the *ras2*Δ strain, which acted as the control for cAMP decline. Under these conditions, CAS-treated cells exhibited a significant decrease in cAMP levels compared to cells that were not exposed to this compound, and the observed effect was close to the effect observed in the *ras2*Δ mutant (Fig. 5a), thus indicating that CAS has a significant impact on cAMP levels.

Cellular cAMP levels are modulated by the competing activities of synthesis through adenylyl cyclase (AC) and degradation mediated by phosphodiesterases Pde1 and Pde2. In this system, AC activity is stimulated by the GTP-binding proteins Ras1 and Ras2, which cycle between a GDP and GTP-bound form. Only the GTP-bound Ras activates AC. To investigate if CAS inhibits PKA and, thereby, affects this aspect of the cAMP-Ras-PKA pathway, we monitored the activation of Ras2 by determining the GTP loading of Ras2 from whole cell lysates^39^. As shown in Fig. 5b, the wild-type strain grown in the presence of CAS displayed reduced levels of Ras2-GTP (~70% reduction in respect to the control cells), similar to the effect caused by glucose starvation. Taking all these results into consideration, we can conclude that CAS modulates cAMP levels by down-regulating the Ras2 function.

**Figure 6:**
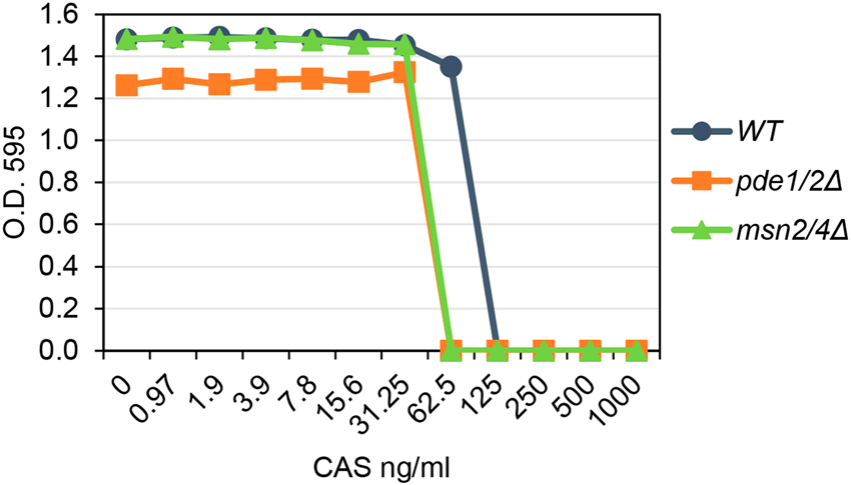
Yeast mutant strains defective in activation of the Slt2-independent genes in response to CAS are sensitive to this compound. CAS sensitivity of the indicated yeast strains was performed in a 96-well microtiter plate assay with CAS concentrations ranging from 1000 to 1 ng/ml. Each well was inoculated with ~10^4^ cells from an exponentially growing culture. Plates were incubated for 48 h at 30 °C, and cell growth was determined by measuring absorbance at 595 nm. The graph corresponds to a representative experiment.

All these results prompted us to study the physiological significance of the PKA inhibition mediated by CAS. To this end, we evaluated CAS sensitivity in mutants defective in activation of the Slt2-independent branch, compared to the wild-type strain. As shown in Fig. 6, strains *pde1*∆*pde2*∆ and *msn2*∆*msn4*∆ displayed a similar moderate sensitivity to CAS in comparison to the behaviour of the wild-type strain. This result supports the notion that PKA inhibition could be beneficial to counteract the CAS effect on the cell wall.

### Caspofungin induces a cytosolic pH decrease dependent on the extracellular domain of the Wsc1 sensor

**Figure 7:**
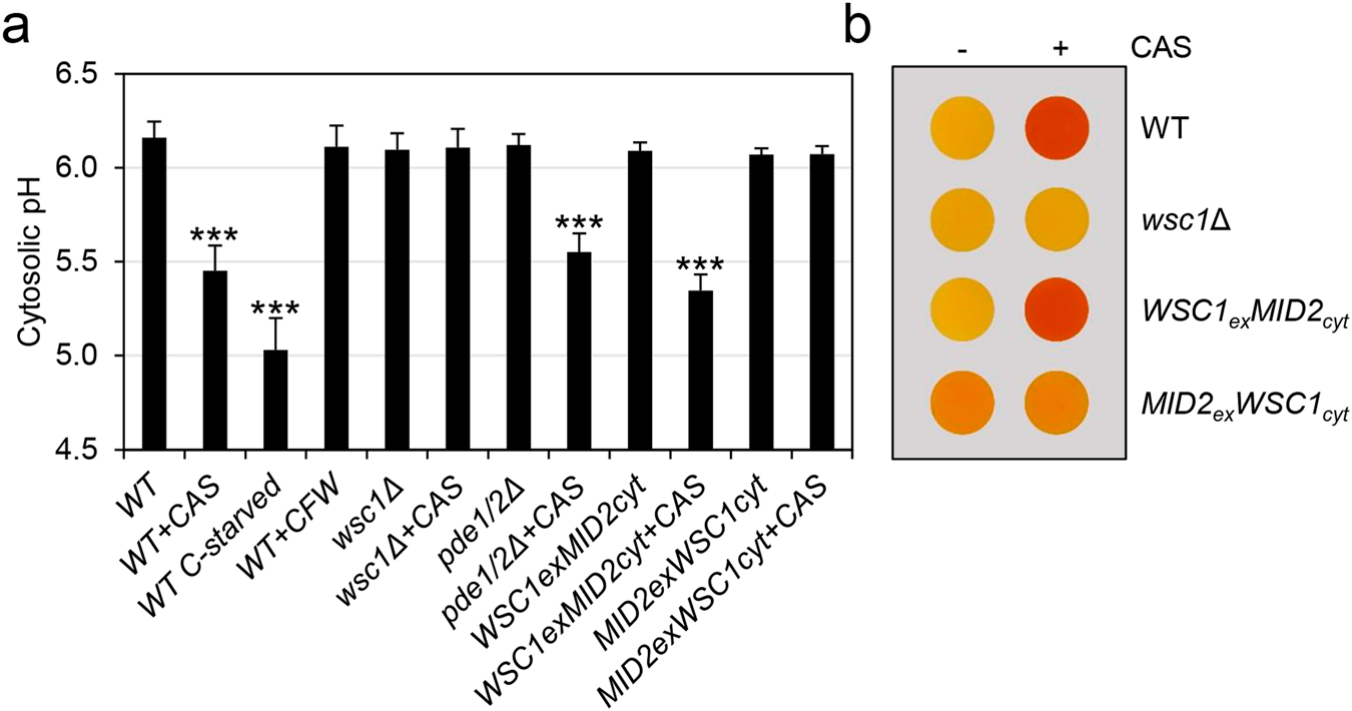
Cytosolic pH is affected by caspofungin treatment. (**a**) Cytosolic pH was determined in the wild-type (WT), wild-type harboring the fusion Wsc1_ex_Mid2_cyt_ or Mid2_ex_Wsc1_cyt_, *wsc1*Δ and *pde1*∆*pde2*∆ cells expressing pHluorin grown in YPD or YPD supplemented with CAS (15 ng/ml, 2 h). Equally, exponential cultures of the WT strain expressing pHluorin were exposed to Calcofluor white in YPD (CFW, 5 μg/ml, 2 h) or glucose starvation. Data are represented as mean and SD of five independent experiments. Statistical analysis of the differences in cytosolic pH was carried out using a two-tailed, unpaired, Student’s t-test: ***p ≤ 0.001. (**b**) Determination of glycogen accumulation, as described in Fig. 4, in the wild-type (WT), wild-type harboring the fusion Wsc1_ex_Mid2_cyt_ or Mid2_ex_Wsc1_cyt_ and *wsc1*Δ strains grown in YPD in the presence or absence of CAS.

Recent research has found that cytosolic pH acts as a cellular signal to activate Ras through the vacuolar ATPase (V-ATPase) in response to glucose availability^26^. We questioned if the negative regulation of Ras2 caused by CAS could be linked to variations in cytosolic pH. To this end, we measured cytosolic pH *in vivo* using a pH-sensitive GFP derivative protein, pHluorin, expressed from a plasmid. This fluorescence ratiometric method allows the measurement of intracellular pH in *S*. *cerevisiae* without requiring staining procedures^40^. Under our growth conditions, the cytosolic pH of the wild-type strain was close to 6.2 in accordance with that previously reported for this genetic background^41^ (Fig. 7a). When the same strain was grown in the presence of CAS, we observed a significant reduction in the cytosolic pH (pH = 5.5), bearing in mind that glucose starvation (severe PKA inhibitory condition) for two hours resulted in a reduction of the cytosolic pH to 5 in wild-type cells. Importantly, in a mutant strain lacking the sensor Wsc1, no variation in cytosolic pH was observed after CAS treatment, as was the case with the wild-type cells exposed to Calcofluor white (Fig. 7a). In order to rule out that the effect on pH reduction was due to a side effect mediated by the PKA inhibition, we measured the cytosolic pH in the presence and absence of CAS in the double mutant *pde1*∆*pde2*∆. This strain, where PKA is activated, showed an identical behaviour to the wild-type strain (Fig. 7a), indicating that pH decrease does not require PKA inhibition.

To gain further insights into the molecular mechanisms involved in the modulation of the cytosolic pH mediated by CAS, we took advantage of two yeast strains, previously used in our lab to study the contribution of the extracellular and cytoplasmic domains of the dominant sensors of the CWI pathway, Wsc1 and Mid2, made to study the process of signal transduction on cell wall stressed cells^13^. These strains contain chimeric versions of the sensors, one expressing the fusion of the extracellular domain of Mid2 to the cytosolic region of Wsc1 (Mid2_ex_Wsc1_cyt_), and the other expressing the fusion of the extracellular domain of Wsc1 to the cytosolic region of Mid2 (Wsc1_ex_Mid2_cyt_). Whereas the first strain shows Slt2 activation and transcriptional induction levels close to the ones of the wild-type strain under CAS treatment, both parameters are completely blocked in cells expressing *MID2*_*ex*_ *WSC1*_*cyt*_^13^. When we studied variations in cytosolic pH in both strains exposed and unexposed to CAS, we found that, in the strain bearing the *WSC1*_*ex*_ *MID2*_*cyt*_ version, CAS induced a pH reduction similar to that observed in the wild-type strain. However, in the case of the strain expressing the fusion *MID2*_*ex*_ *WSC1*_*cyt*_, pH reduction was completely blocked, mimicking the situation described for a *wsc1*Δ mutant (Fig. 7a). Moreover, these results were in agreement with the increment in glycogen accumulation induced by CAS in the strain bearing the extracellular region of Wsc1 and its severe reduction when the extracellular region of Mid2 is present (Fig. 7b). These results indicate that the extracellular domain of Wsc1 is required not only for Slt2 activation and the corresponding upregulation of the Slt2-dependent gene expression but also to produce the inhibitory effect on the cAMP/PKA pathway triggered by CAS.

## Discussion

Echinocandins are the most recent addition to the antifungal arsenal, and they have become essential in the prophylaxis and treatment of invasive infections caused by *Candida* and *Aspergillus* species. The mechanism of action of these drugs involves the inhibition of the β-1,3-glucan synthase, an enzyme complex that is located in the plasma membrane of fungal cells^42, 43^. Since the β-1,3-glucan polymer is a key structural component of the fungal cell wall, inhibition of its synthesis causes loss of cell wall integrity and cell wall stress. Over the last few decades, studies using yeast models have been very useful for enhancing understanding of how signal transduction pathways work. In this regard, *Saccharomyces cerevisiae* elicits an adaptive response to cell wall stress to overcome damage to this essential structure. This response is mainly mediated by the cell wall integrity MAPK signalling cascade governed by the MAPK Slt2 and the transcription factor Rlm1. The activation of Slt2 triggers Rlm1 phosphorylation, which consequently interacts with the chromatin remodelling complex SWI/SNF to direct its association with the specific binding sites at the promoters of the genes transcriptionally induced upon CWI pathway activation^9, 44^. Accordingly, CAS provokes the activation of Slt2, triggering the corresponding transcriptional response^11–13^. Both, Slt2 hyperphosphorylation^11, 13^ and the indicated transcriptional response^13^ are dependent on the CWI pathway sensor Wsc1. In this work, we further characterized the regulation of the transcriptional profile elicited by CAS treatment of yeast cells, remarkably showing that the MAPK Slt2 and the transcription factor Rlm1 only control a set of upregulated genes, mainly those genes associated with different cell wall stress conditions. Additionally, we discovered a second large group of genes that are functionally associated with the nutrient-regulated cAMP/PKA pathway, one of the major pathways that transduce nutrient signals to regulate cell growth, in addition to TOR and Sch9 pathways^22, 23^. Specifically, this group of Slt2-independent transcripts correspond to genes that exhibit upregulated expression in conditions where the cAMP/PKA pathway is deactivated, as occurs during the stationary phase of growth or under situations of carbon deprivation^22, 23, 33^. In fact, constitutive activation of PKA abrogates the gene induction of this branch of the transcriptional response elicited by CAS. Global transcriptional profiles of yeast cells treated with CAS have been previously reported^11, 12^. Here, comparison of the transcriptional profiles of the wild-type strain and the *slt2*∆ mutant, in combination with MARQ data-mining, have allowed us uncover a novel regulatory mechanism associated to PKA inhibition not previously described.

In terms of the PKA-related transcriptional response caused by CAS treatment, only a limited group of genes was dependent on Msn2/4 transcription factors, despite the high number of genes bearing STRE elements in their promoter region. This supports that other regulatory elements should participate in a cooperative manner. In fact, the transcription factor Rph1 has been implicated in the STRE-mediated activation of gene expression after the diauxic shift (glucose depletion) and early stationary phase, regulating gene expression synergistically or redundantly with the transcription factor Gis1^29^. Moreover, this transcriptional regulatory network becomes more complex in light of the fact that a large set of genes has also been described to be regulated cooperatively by Gis1 and Msn2/4 at the diauxic transition^45^.

Previous studies on yeast have also reported interactions between the CWI and the cAMP/PKA pathways with different elements being involved in each pathway. For example, in a phenotypic study, the Wsc1 sensor was proposed to act downstream of Ras proteins under heat shock stress^46^, and the role of the Rho1 GTP/GDP exchange factor Rom2 in Ras-cAMP down-regulation in response to oxidative stress has also been described^47, 48^. Notably, our studies on CAS points to a new type of interaction taking place upstream of Ras2 and independently of Rom2. Additionally, the significant complexity of the interconnection between these pathways is highlighted by the fact that, under certain circumstances, the CWI MAPK Slt2 phosphorylates the PKA regulatory subunit Bcy1 through the protein kinase Sch9 in response to TORC1 inhibition^49, 50^. However, the inhibitory effect of CAS on the PKA pathway is not affected by the absence of Slt2.

Remarkably, other stresses, like heat shock^46^, oxidative stress^47, 48^, alkaline pH^51^, and stress of the endoplasmic reticulum^52^, have been postulated to inhibit the PKA pathway. In all cases, the mechanistic details of how these stresses impact on the PKA activity are not fully understood. Here, we show that exposure of yeast cells to CAS leads to events related to the inhibition of PKA activity, which would explain why the expression levels of genes that are negatively regulated by the PKA are upregulated. Additionally, CAS exposure triggers a decrease in cytosolic pH.

Recent data indicates that cytosolic pH variation results from the glucose metabolism acting as a cellular signal that modulates the activation of Ras/PKA through the vacuolar ATPase^26^, linking glucose metabolism to the regulation of cell growth. The fact that CAS induces a reduction of the cytosolic pH could imply the existence of some type of interaction at this level. However, it is unlikely that CAS inhibits Ras activity affecting negatively to the glucose metabolism resulting in a decrease in the cytosolic pH, since the associated transcriptional response relies on the extracellular region of the sensor Wsc1. This sensor functions as a mechanosensor, specifically detecting the cell surface stress caused by alterations to the glucan network^53^. In addition to its role as a signal that regulates cell growth in response to nutrients, it has been hypothesized that cytosolic pH may also integrate other environmental signals and stresses via multiple mechanisms. Thus, modulation of the Ras/PKA activity would consist of a common readout of specific stresses that might contribute to cellular adaptation. This notion is further supported by the fact that oxidative stress induced by the addition of H_2_O_2_ rapidly reduces cytosolic pH^54^. Our results are in agreement with this idea, since cell wall stress mediated by CAS also reduces cytosolic pH in a Wsc1-dependent manner. This drop in pH does not appear a secondary effect of PKA inhibition, since in the *pde1*∆*pde2*∆ mutant the pH reduction is still observed. However, we cannot rule out that alternative unknown mechanisms could be involved.

The inhibitory effect of CAS on the PKA activity, measured *in vivo* using the PKA substrate Pat1, correlates with the temporal profile of cell wall stress responses mediated by the CWI pathway, reaching a peak of Slt2 phosphorylation after one to three hours of treatment. Specifically, the time-course analysis of the PKA-dependent phosphorylation of the Pat1 reporter indicated that phosphorylation was almost completely lost not before 90 minutes of exposure to CAS, while the PKA-dependent phosphorylation of this substrate is rapidly lost (in less than 10 minutes) upon glucose starvation^37^. In addition, the kinetics of Msn2 nuclear translocation after CAS treatment is also similar to that of the CWI pathway activation. Interestingly, in contrast to other stress responses, the nuclear localisation of Msn2 occurred only in a fraction of the cell population and more delayed in time. This effect is compatible with the observation that different stresses elicit qualitatively different dynamics of Msn2 nuclear translocation^55^. Therefore, cells would modulate different dynamical parameters of Msn2 nuclear translocation in response to changes in intensity of different stresses, probably related to different patterns of target gene expression. For example, intensity variations of glucose limitation or osmotic stress elicit duration modulation of the initial peak, but in the case of oxidative stress induces primarily amplitude modulation of nuclear localisation^55^. In this context, the cell wall damage caused by the concentration of CAS used in this work would elicit a specific pattern of Msn2 subcellular localisation.

In terms of the biological relevance of the PKA inhibition under cellular stress conditions, we have determined that yeast strains where the Slt2-independent branch of response to CAS is affected, such as *msn2*∆*msn4*∆ or *pde1*∆*pde2*∆, are more sensitive to this compound than a wild-type strain. Moreover, it is worth mentioning that PKA activity inhibits the formation of the processing bodies (P-bodies) induced by a variety of stress conditions, like hyperosmotic or oxidative stress^56^. In fact, constitutive PKA signalling in the presence of the dominant *RAS2* ^val19^ allele inhibits P-body formation^56^. P-bodies consist of cytoplasmic ribonucleoprotein foci that are found in all eukaryotes, from yeast to humans, and are related to the response to stress implicated in mRNA processing and translation^57^. In accordance with this, we have observed that CAS also induces the formation of P-bodies in a PKA-dependent manner (our unpublished results), which could be related to the inhibitory effect of CAS on the PKA activity.

The involvement of cAMP-PKA signalling in environmental sensing and growth is well conserved across the fungal kingdom. Basic research on PKA-mediated processes, such as those accomplished in this work, is complicated due to the pleiotropic nature of this pathway; however, this pleiotropy has been postulated to become this pathway an ideal candidate for antifungal intervention^58^. Indeed, a tight control of PKA signalling is required for a full virulence phenotype of the three major fungal pathogens: *Candida albicans*, *Cryptococcus neoformans* and *Aspergillus fumigatus*, all of which globally affect morphogenesis control (e.g., filamentation or spore germination), resistance to host defences (e.g., capsule formation or melanin biosynthesis), and metabolic adaptation or stress responses^58^. In addition, it has recently been demonstrated a direct connection between the CWI and cAMP/PKA pathways in the regulation of intracellular cAMP levels in *Cryptococcus neoformans* ^59^. All these data reinforce the notion that combinational therapy, affecting PKA regulation in parallel to another relevant physiological process (e.g., compounds that interfere with the cell wall integrity), could be a valuable strategy to combat the rising threat of antifungal drug resistance.

## Methods

### Yeast strains and plasmids

All of the experiments were performed with the *S*. *cerevisiae* BY4741 strain (*MAT*a, *his3*Δ*1*, *leu2*Δ*0*, *met15*Δ*0*, *ura3*Δ*0*) and mutant derivatives provided by Euroscarf (Frankfurt, Germany). Single mutant strains present the corresponding gene completely deleted and replaced by the geneticin resistance-codifying *Kan*MX4 module. The yeast strains from this collection used in this work were: *wsc1*Δ, *rom2*Δ, *bck1*Δ, *slt2*Δ, *rlm1*Δ, *ras2*Δ and *rim15*Δ. To generate the *bcy1*Δ and *pde1*Δ *pde*2Δ mutant strains, the *BCY1* and *PDE2* genes were replaced by *KanMX4 and HIS3MX6* modules respectively, in the corresponding wild-type BY4741 and *pde1*Δ strains using the SFH PCR-based method as previously described^60^. The double mutant strain *msn2*Δ*msn*4Δ (RG001) and strains BAS3 (*wsc1*::*SpHIS5 mid2*::*MID2*_*ex*_ *WSC1*_*cyt*_) and BAS4 (*mid2*::*KlLEU2 wsc1*::*WSC1*_ex_ *MID2*_cyt_) expressing the chimeric constructions of Wsc1 and Mid2 have been previously described^13, 61^. The plasmids used in this work were p*ADH1*-*MSN2*-GFP^34^ provided by Dr. Christoph Schüller (Institute of Applied Genetics and Cell Biology, University of Resources and Life Science, Vienna, Austria); pRS423-pr^*CUP*^-*6xMYC*-*cki1* ^*2*-*200*(*S125/130A*)36^ provided by Dr. Jodi Nunnari (Department of Molecular and Cellular Biology, University of California, Davis, USA); pTS118, which contains a constitutively active *RAS2* (*RAS2* ^val19^ allele) cloned in YCplac33 plasmid^38^ provided by Dr. Michael N. Hall (Division of Biochemistry, Biozentrum, University of Basel, Switzerland); the expression vector pGEX2T-RBD, provided by Dr. A. Wittinghofer (Max-Planck Institute, Dortmund, Germany); pPHY3362, which contains the *PAT1*pro-Myc-Pat1 construction^37^ provided by Dr. Paul K. Herman (Department of Molecular Genetics, The Ohio State University, Columbus, USA); and, finally, the pHI-U (*URA3* marker) plasmid provided by Dr. Hana Sychrová (Academy of Sciences of the Czech Republic, Prague, Czech Republic), in which expression of a pH-sensitive variant of the green fluorescent protein, pHluorin, is under the control of the *ADH1* promoter^41^.

### Growth conditions

Yeast cells were routinely grown overnight in a YPD medium of 2% glucose, 2% peptone (Cat. Number 1616, Conda, Madrid, Spain), and 1% yeast extract (Cat. Number 103753, Merck Millipore, Darmstadt, Germany), at 220 rpm and 24 °C to an optical density of 0.8–1 (A_600_). The culture was refreshed to 0.2 (A_600_) in YPD and grown for 2 h and then divided into two parts. One part was allowed to continue growing under the same conditions (the non-treated culture), and the other was supplemented with caspofungin (kindly provided by Merck Sharp and Dohme [MSD] Research Laboratories, USA), aminocandin (an investigational echinocandin, kindly provided by Indevus Pharmaceuticals, USA), both at a final sublethal concentration of 15 ng/ml, or Calcofluor white (CFW) (Fluorescent Brightener 28, Sigma-Aldrich, USA) at 5 µg/ml. The cells were collected and subsequently processed according to the experimental approach, as described below. For carbon starvation studies, exponentially growing cells in YPD were collected by centrifugation and then washed twice with a medium lacking glucose (YP or SC-D). Finally, cells were resuspended in YP or SC-D and incubated for the indicated times. SC medium (0.17% yeast nitrogen base, 0.5% ammonium sulfate, 2% glucose, and the required amino acids) was used to select the yeast cells transformed with plasmids. CAS sensitivity (MIC) assays were performed as previously described^16^.

### Microarray experiments

Total RNA isolation and purification was carried out as previously described^18^. Genome-wide transcriptional profiles were obtained using Affymetrix GeneChip Yeast Genome 2.0 arrays (Affymetrix, Santa Clara, CA). cDNA synthesis and microarray hybridization, image analysis, data processing, and statistical analysis were carried out as described^62^. The files generated from the scanning (.CEL) were converted to gene expression signals using the RMA algorithm included in the Affymetrix Expression Console. For all genes analysed, fold changes between the experimental conditions under comparison were calculated as a quotient between the mean of the gene expression signals. The expression of each gene was considered to be up- or downregulated when the expression ratio under the conditions tested was ≥2 or ≤0.5 respectively. Three independent biological samples for each condition were processed and analysed. Statistical analysis was performed with Student’s t-test. Values of *p* < 0.05 were considered to be significant, and the corresponding genes underwent further analysis.

To determine whether the gene induction observed in the wild-type (WT) strain after caspofungin treatment (15 ng/ml, 2 h) was significantly reduced in *slt2*Δ, *rlm1*Δ, and *msn2*Δ*msn4*Δ mutant strains, we assessed the relationship between the responses of each mutant (mutant ratio) versus those of the WT strain (WT ratio). A mutant ratio/WT ratio value of 0.65 was considered the threshold for defining a significant reduction in gene induction^63^. In any event, the genes that exhibited an expression ratio in the mutant of <1.6 were not deemed to be upregulated. The data obtained were analysed using MARQ^30^, a web-based application that enables users to query signatures derived from GEO and retrieve experimental conditions that may induce similar or opposite gene expression programs to a given query (in this case, transcriptional response to CAS treatment).

### Quantitative real-time PCR assays

Quantitative RT-PCR (RT-qPCR) assays were performed as described^18^. To quantify gene expression, the abundance of each transcript was determined relative to the standard transcript of *ACT1* for input cDNA normalization, and the final data on relative gene expression between the conditions tested (treated vs. untreated sample) were calculated following the 2^−∆∆Ct^ method^64^. Primer sequences are available upon request.

### Fluorescence microscopy

For Msn2-GFP localisation studies, exponentially growing cells transformed with the p*ADH1*-*MSN2*-GFP plasmid treated with CAS (15 ng/ml) or CFW (5 µg/ml) for two hours were directly visualized and analysed by fluorescence microscopy and differential interference contrast (DIC) using a Nikon Eclipse TE2000-U fluorescence inverted microscope equipped with CCD. Digital images were acquired with an Orca C4742-95-12ER camera (Hamamatsu Photonics, Japan) and processed with the Hamamatsu HCImage imaging system software. Nuclei were stained by addition of 10 µg/ml of DAPI to the cultures 10 min before microscopy.

### Western blotting and immunoprecipitation assays

Immunoblot analyses, including cell collection and lysis, fractionation of proteins by SDS-PAGE, and transfer to nitrocellulose membranes were carried out as previously described^61^. The composition of the lysis buffer was as follows: 50 mM Tris-HCl (pH 7.5), 10% glycerol, 1% Triton X-100, 0.1% SDS, 150 mM NaCl, 0.1% NP-40, 5 mM EDTA (pH 8), 2.1 mg/ml NaF, 0.18 mg/ml sodium orthovanadate, 10.8 mg/ml glycerol 2-phosphate, 2.3 mg/ml disodium pyrophosphate, 1 mM PMSF and protease inhibitor cocktail (Roche). The ECL detection system or the Odyssey Infrared Imaging System (LI-COR Biosciences) was used depending on the experimental approach. 40 ml of the exponentially growing cells transformed with the pRS423-pr^*CUP*^-*6xMYC*-*cki1* ^*2*-*200*(*S125/130A*)^ plasmid and treated or untreated with CAS (15 ng/ml) were collected for Western blotting. Fusion Myc-Cki1 was detected using the anti-c-Myc monoclonal antibody (clone 9E10, Cat. Number: 626801, BioLegend, USA). The primary antibody was detected using the IRDye 680LT goat anti-mouse antibody from LI-COR. This Cki1 variant contains the first 200 amino acids of Cki1 and possesses mutations at two known protein kinase C sites (S125A, S130A) making its phosphorylation (as detected by a mobility shift in SDS-PAGE) exclusively PKA dependent (S185)^36^.

To monitor the levels of PKA-dependent phosphorylation of Pat1, cell extracts for immunoprecipitation (at different time intervals of CAS treatment) were prepared by resuspending log phase wild-type cells transformed with the plasmid *PAT1*pro-Myc-Pat1 (expressing the Myc-Pat1 fusion from the native *PAT1* promoter) in lysis buffer (as indicated above, but lacking Triton X-100 and SDS) and lysing by disruption with glass beads in a Fastprep instrument (Fastprep-24, MP Biomedicals, USA). Myc-tagged Pat1 was immunoprecipitated with the anti-c-Myc monoclonal antibody at 4 °C overnight, and the immunoprecipitates were collected on rProtein A Sepharose Fast Flow (17-1279-01, GE Healthcare Life Sciences) for 90 min at 4 °C. After undergoing extensive washing with lysis buffer and boiling in 5x SDS loading buffer, the level of PKA phosphorylation in the immunoprecipiated material was assessed using the anti-Phospho-PKA substrate monoclonal antibody (Cat. Number 9624, Cell Signaling Technology, USA). Total Myc-Pat1 levels were determined using the anti-c-Myc monoclonal antibody.

Image Studio Lite 5.0 software from LI-COR Biosciences was used to determine the signal intensity of the bands on the Western blots.

### Glycogen staining

Glycogen content was determined following the staining protocol previously described^65^ with some modifications. For each determination, 30 ml of exponentially growing cultures were harvested by centrifugation at 2,500 rpm at room temperature for 3 min. After that, cells were resuspended in 1 ml of a solution of 0.2% iodine/0.4% potassium iodide. After 3 min at room temperature, the cells were centrifuged, resuspended in 30 µl of the same solution, and spotted onto agar plates. Photographs were immediately taken, and the corresponding images were subsequently processed with Adobe Photoshop CS6.

### Measurement of cAMP levels

The cAMP enzyme immunoassay (EIA) system kit from GE Healthcare Life Sciences (RPN2251) was used to determine the intracellular levels of cAMP. For each condition tested, 30 ml of exponentially growing cultures was harvested by centrifugation at 2,500 rpm at 4 °C for 3 min and cells were immediately frozen in liquid nitrogen. Next, the cells were thawed on ice and washed with cold PBS before the wet weight was determined. The cells were subsequently resuspended in 350 µl of lysis reagent 2B, included in the kit, and 150 µl of glass beads were added. Each suspension was vortexed for 30 min at 4 °C and then spun down at 12,000 rpm for 10 min at 4 °C. Finally, 100 µl of supernatant, in duplicate, was used to measure concentrations of intracellular cAMP using the non-acetylation protocol described in the kit. Three independent experiments were carried out for each strain and condition tested.

### Determination of the Ras2-GTP/Total Ras2 ratio *in vivo*

Determination of the Ras2-GTP level was performed by GST-RBD pull-down assay that has been described previously^66^. GST-RBD was obtained from *Escherichia coli* BL21 cells carrying the plasmid pGEX2T-RBD, which encodes amino acids 1–149 of Raf-1 (Ras-binding domain) fused to GST. For the pull-down assay, the yeast whole cell extract was incubated with GST-RBD-bound glutation beads for 4 h. After washing, bound proteins were eluted with sample buffer and analysed by Western blotting with anti-Ras2 (yC-19) polyclonal antibody (sc-6759, Santa Cruz, USA) using the Odyssey Infrared Imaging System from LI-COR. The primary antibody was detected using the IRDye 800CW donkey anti-goat antibody from LI-COR. To determine the signal intensity of the bands, the Image Studio Lite 5.0 software from LI-COR Biosciences was used.

### Measurement of cytosolic pH

Cytosolic pH measurements were performed following methods previously described^40, 41^. Briefly, to study the effect of the presence or absence of CAS (15 ng/ml) and CFW (5 µg/ml) over 2 h in YPD medium, log phase cultures of cells expressing pHluorin (transformed with plasmid pHI-U) and control cells lacking the plasmid were centrifuged for 3 min at 3,000 rpm and washed twice with ice-cold PBS. In addition, for glucose deprivation conditions, cells were grown in YPD medium and transferred to the SC-D for 2 h before being processed as per the method described above. To measure cytosolic pH, the same amounts of cells were resuspended in 500 μl of citrate-phosphate (CP) buffer pH 6 (~1.5 A_600_). In parallel, to obtain the calibration curve for each experiment, wild-type cells expressing pHluorin were resuspended in 500 μl of a series of calibration buffers (50 mM MES, 50 mM HEPES, 50 mM KCl, 50 mM NaCl, 200 mM ammonium acetate, 10 mM NaN_3_, 10 mM 2-deoxyglucose) adjusted to pH 5.0, 5.5, 6.0, 6.5, 7.0, 7.5, and 8.0, and incubated at 30 °C for 30 min. 100 μl of each cell suspension (calibration or experimental samples) were transferred to a 96-well flat-bottom microtiter plate (Nunc, Denmark) in quadruplicate. pHluorin fluorescence emission at 530 nm after excitation to 380 nm (*I*_*380*_) and 485 nm (*I*_*485*_) was acquired using a FL600 microplate fluorescence reader (Bio-Tek Instruments, Inc. USA). All the fluorescence intensity signals were background-subtracted using data from the untransformed, pHluorin-free samples. A calibration curve of the ratio *I*_*380*_/*I*_*485*_ versus pH was obtained and fitted to a third-degree polynomial regression using the SigmaPlot 11.0 (Systat Software, Inc., USA) software. To estimate cytosolic pH from experimental samples, *I*_*380*_/*I*_*485*_ ratios were used to obtain pH values according to the calibration curve. All pH determination experiments were repeated at least five times (biological replicates).

### Data Availability

The microarray data described in this work follows the recommendations pertaining to the minimum information about a microarray experiment and have been deposited at the National Center for Biotechnology Information gene expression and hybridization array data repository with accession number GSE80394.

## Electronic supplementary material

Supplementary Information
