# Supplementary material for: A novel connection between the Cell Wall Integrity and the PKA pathways regulates cell wall stress response in yeast
Source: Sci Rep. 2017 Jul 18;7:5703. doi: 10.1038/s41598-017-06001-9 (PMC5515849; doi:10.1038/s41598-017-06001-9)
Supplement: Supplementary file 1 — Supplementary Information [file 41598_2017_6001_MOESM1_ESM.pdf]

A novel connection between the Cell Wall Integrity and the PKA pathways regulates cell wall stress response in yeast

SUPPLEMENTAL INFORMATION

Raúl García, Enrique Bravo, Sonia Diez-Muñiz, Cesar Nombela, Jose M. Rodríguez-Peña and Javier Arroyo

Table S1

ORF	Gene	WT CAS	WT AMC	CR-ZY	*slt2* Δ	*rlm1* Δ		*msn2/4* Δ		Functional group	Description
YPR160W	*GPH1*	6.8	9.4		3.7	8.5		3.0	•	carbohydrate metabolism	Glycogen phosphorylase required for the mobilization of glycogen
YPL088W	---	5.7	11.3	•	2.1	1.6	•	5.9		carbohydrate metabolism	Putative aryl alcohol dehydrogenase
YJL155C	*FBP26*	2.5	2.8	•	1.2	1.3	•	2.4		carbohydrate metabolism	Fructose-2,6-bisphosphatase
YIL107C	*PFK26*	2.0	2.2		1.2	1.3	•	1.9		carbohydrate metabolism	6-phosphofructo-2-kinase
YHR043C	*DOG2*	2.0	1.5		1.0	1.4	•	1.6		carbohydrate metabolism	2-deoxyglucose-6-phosphate phosphatase
YKL163W	*PIR3*	18.8	47.3	•	0.5	1.0	•	19.2		cell wall	O-glycosylated covalently-bound cell wall protein
YKR091W	*SRL3*	7.3	10.4	•	1.3	1.2	•	5.4		cell wall	GTB motif (G1/S transcription factor binding) containing protein
YIR039C	*YPS6*	3.2	3.3	•	1.3	1.5	•	2.5		cell wall	Putative GPI-anchored aspartic protease
YKR061W	*KTR2*	3.0	4.0		1.4	1.1	•	2.7		cell wall	Mannosyltransferase involved in N-linked protein glycosylation
YDR055W	*PST1*	2.9	5.8	•	0.5	0.8	•	2.5		cell wall	Cell wall protein that contains a putative GPI-attachment site
YLR121C	*YPS3*	2.9	4.2	•	1.1	1.3	•	2.3		cell wall	Member of family of proteases involved in cell wall maintenance
YJL160C	*PIR5*	2.5	2.9		1.0	1.4	•	2.2		cell wall	Member of the PIR family of cell wall proteins
YKL104C	*GFA1*	2.4	2.5	•	0.7	1.0	•	2.5		cell wall	Glutamine-fructose-6-P amidotransferase of chitin biosynthesis
YNL192W	*CHS1*	2.1	2.8	•	1.4	1.5	•	2.0		cell wall	Chitin synthase I
YGR189C	*CRH1*	2.1	2.7	•	0.9	1.2	•	1.9		cell wall	Chitin transglycosylase; transfer of chitin to beta(1-6) and (1-3) glucans
YDR077W	*SED1*	2.0	2.3	•	1.2	1.3	•	1.8		cell wall	Major stress-induced structural GPI-cell wall glycoprotein
YDL181W	*INH1*	2.3	3.0		1.6	2.3		2.6		generation of energy	Protein that inhibits ATP hydrolysis by the F1F0-ATP synthase
YHR209W	*CRG1*	9.5	16.1	•	1.5	1.7	•	8.7		lipid metabolism	S-AdoMet-dependent methyltransferase involved in lipid homeostasis
YPL110C	*GDE1*	2.2	2.2		1.1	1.1	•	2.0		lipid metabolism	Glycerophosphocholine (GroPCho) phosphodiesterase
YJL108C	*PRM10*	6.0	6.7	•	3.5	3.8	•	4.8		mating	Pheromone-regulated protein
YIL117C	*PRM5*	4.3	6.7	•	1.1	1.0	•	3.3		mating	Pheromone-regulated protein
YEL059C-A	*SOM1*	2.1	1.6		1.4	1.3	•	1.7		mitochondrial	Subunit of the mitochondrial inner membrane peptidase
YMR238W	*DFG5*	2.0	2.0	•	1.2	1.1	•	1.9		morphogenesis	Putative mannosidase
YPL052W	*OAZ1*	2.0	2.3		1.1	1.1	•	2.1		protein modification	Regulator of ornithine decarboxylase Spe1
YFL014W	*HSP12*	13.3	25.9	•	6.7	16.7		28.5		response to stress	Protein involved in maintaining membrane organization
YMR175W	*SIP18*	10.5	9.0		4.2	5.7	•	2.7	•	response to stress	Phospholipid-binding hydrophilin
YOL052C-A	*DDR2*	7.0	9.4		3.6	6.5		3.8	•	response to stress	Multi-stress response protein
YPR005C	*HAL1*	2.5	3.8	•	1.3	1.7		2.6		response to stress	Cytoplasmic protein involved in halotolerance
YCR104W	*PAU3*	2.3	2.2		1.5	1.8		3.0		response to stress	Member of the seripauperin multigene family
YBR203W	*COS111*	2.2	2.7		1.5	2.0		1.7		response to stress	Protein required for antifungal drug ciclopirox olamine resistance
YDR533C	*HSP31*	2.2	2.2		1.5	1.8		1.9		response to stress	Methylglyoxalase that converts methylglyoxal to D-lactate
YKL161C	*KDX1*	14.9	41.4	•	1.0	1.0	•	11.5		signal transduction	Protein kinase of the cell wall integrity pathway
YDR085C	*AFR1*	5.6	9.8	•	1.6	1.6	•	3.9		signal transduction	Protein required for pheromone-induced projection formation
YMR104C	*YPK2*	3.6	4.6		1.0	1.0	•	3.5		signal transduction	Protein kinase similar to serine/threonine protein kinase Ypk1

YGR023W	*MTL1*	2.7	2.7		1.3	1.5	•	2.2		signal transduction	Plasma membrane sensor involved in cell integrity signaling
YOR208W	*PTP2*	2.7	3.2	•	1.3	1.2	•	2.5		signal transduction	Phosphotyrosine-specific phosphatase involved in osmosensing
YHR030C	*SLT2*	2.7	3.5	•	1.2	1.1	•	2.4		signal transduction	Serine/threonine MAP kinase of cell wall integrity pathway
YCR073C	*SSK22*	2.3	3.0		1.0	0.8	•	2.2		signal transduction	MAP kinase kinase kinase of the HOG1 pathway
YPL089C	*RLM1*	2.2	2.5		1.1	1.0	•	1.8		signal transduction	MADS-box transcription factor activated by the MAP-kinase Slt2p
YGL248W	*PDE1*	2.0	2.0		1.4	1.4	•	1.6		signal transduction	Low-affinity cyclic AMP phosphodiesterase
YHL022C	*SPO11*	2.5	2.2		1.3	1.6	•	2.3		sporulation	Meiosis-specific protein that initiates meiotic recombination
YNL294C	*RIM21*	2.2	2.3	•	1.0	1.0	•	1.9		sporulation	pH sensor molecule, component of the RIM101 pathway
YDR277C	*MTH1*	2.2	3.2		1.5	2.3		1.6		transcription	Negative regulator of the glucose-sensing signal transduction pathway
YCL069W	*VBA3*	4.5	2.1		1.4	1.2	•	4.8		transport	Permease of basic amino acids in the vacuolar membrane
YIL023C	*YKE4*	3.0	2.4	•	1.0	1.1	•	2.4		transport	Zinc transporter; localizes to the ER
YDR342C	*HXT6*	2.9	3.9		1.6	2.0		1.3	•	transport	High-affinity glucose transporter
YNR065C	---	2.9	3.4		1.1	0.8	•	2.8		transport	Protein of unknown function
YNL293W	*MSB3*	2.7	2.1		1.0	1.2	•	2.2		transport	Rab GTPase-activating protein; regulates endocytosis
YAL053W	*FLC2*	2.4	2.6	•	1.0	1.1	•	2.2		transport	Putative calcium channel
YNL015W	*PBI2*	2.2	2.4		1.6	2.1		2.1		transport	Cytosolic inhibitor of vacuolar proteinase B (PRB1)
YNR066C	---	2.2	2.7		0.7	0.8	•	1.5	•	transport	Putative membrane-localized protein of unknown function
YOR306C	*MCH5*	2.1	2.7	•	0.9	0.7	•	2.1		transport	Plasma membrane riboflavin transporter
YHR092C	*HXT4*	2.1	3.6		1.2	1.9		1.8		transport	High-affinity glucose transporter
YGR166W	*TRS65*	2.1	2.4		0.9	1.0	•	2.2		transport	Component of transport protein particle (TRAPP) complex II
YDR070C	*FMP16*	7.0	10.2		3.8	6.0		2.8	•	unknown	Protein of unknown function
YJL107C	---	6.0	6.1	•	2.5	3.7	•	2.3	•	unknown	Putative protein of unknown function
YJL161W	*FMP33*	3.6	4.9	•	1.6	2.2	•	3.6		unknown	Putative protein of unknown function
YOL159C	---	3.5	4.0		1.2	1.2	•	3.1		unknown	Soluble protein of unknown function
YPR078C	---	3.5	3.5		1.3	1.0	•	3.5		unknown	Putative protein of unknown function
YFL061W	*DDI2*	3.0	1.9		1.4	1.8	•	2.9		unknown	Protein of unknown function
YNL058C	---	2.9	4.2	•	0.7	1.2	•	2.4		unknown	Putative protein of unknown function
YIL029C	---	2.4	2.1		1.6	1.8		2.6		unknown	Putative protein of unknown function
YBL049W	*MOH1*	2.3	1.8		1.5	2.4		2.2		unknown	Protein of unknown function
YCR099C	---	2.3	2.2		1.2	1.5	•	2.7		unknown	Protein of unknown function
YPL067C	---	2.2	2.8		1.1	1.4	•	2.0		unknown	Putative protein of unknown function
YJL103C	*GSM1*	2.2	2.8		1.6	2.2		2.6		unknown	Putative zinc cluster protein of unknown function
YIL108W	---	2.1	2.6	•	1.1	1.1	•	2.3		unknown	Putative metalloendopeptidase
YCL049C	---	2.1	2.1	•	1.1	1.1	•	1.7		unknown	Protein of unknown function
YGL258W-A	---	2.1	2.1		1.5	1.5	•	1.8		unknown	Putative protein of unknown function
YGR149W	---	2.0	2.3	•	1.4	1.6		1.9		unknown	Putative protein of unknown function
YDL057W	---	2.0	1.9		1.3	1.8		1.8		unknown	Putative protein of unknown function

YNL115C	---	2.0	2.0		1.3	1.5	•	1.9		unknown	Putative protein of unknown function
YLR142W	*PUT1*	6.6	10.7		11.8	12.2		6.2		amino acid metabolism	Protein involved in utilization of proline as sole nitrogen source
YBR117C	*TKL2*	6.3	10.4		4.7	6.7		1.6	•	amino acid metabolism	Transketolase
YDR380W	*ARO10*	5.6	6.3		8.3	9.7		3.9		amino acid metabolism	Phenylpyruvate decarboxylase
YJL088W	*ARG3*	2.7	5.0		3.4	4.7		2.1		amino acid metabolism	Ornithine carbamoyltransferase
YGL184C	*STR3*	2.7	3.0		5.1	6.2		3.0		amino acid metabolism	Peroxisomal cystathionine beta-lyase
YHR137W	*ARO9*	2.6	2.3		2.7	3.0		2.3		amino acid metabolism	Aromatic aminotransferase II
YMR250W	*GAD1*	2.6	3.4		2.6	3.1		2.1		amino acid metabolism	Glutamate decarboxylase
YJR078W	*BNA2*	2.5	3.2		3.0	3.7		1.3	•	amino acid metabolism	Tryptophan 2,3-dioxygenase or indoleamine 2,3-dioxygenase
YKR076W	*ECM4*	2.3	3.0		2.5	2.4		1.8		amino acid metabolism	Omega class glutathione transferase
YNL277W	*MET2*	2.3	1.8		3.2	3.1		2.9		amino acid metabolism	L-homoserine-O-acetyltransferase
YJL153C	*INO1*	13.3	41.3		14.8	12.9		18.2		carbohydrate metabolism	Inositol 1-phosphate synthase
YGR256W	*GND2*	5.5	6.4		4.6	6.5		3.1	•	carbohydrate metabolism	6-phosphogluconate dehydrogenase
YGR248W	*SOL4*	4.1	4.8	•	3.2	4.6		1.5	•	carbohydrate metabolism	6-phosphogluconolactonase
YGL156W	*AMS1*	3.0	4.2		2.9	3.6		2.7		carbohydrate metabolism	Nannosidase involved in free oligosaccharide (fOS) degradation
YMR105C	*PGM2*	2.8	4.3	•	2.6	2.7		1.9		carbohydrate metabolism	Catalyzes the conversion from glucose-1-P to glucose-6-P
YEL011W	*GLC3*	2.6	2.8		3.0	3.2		2.1		carbohydrate metabolism	Glycogen branching enzyme, involved in glycogen accumulation
YFR053C	*HXK1*	2.6	3.4		1.7	2.5		2.2		carbohydrate metabolism	Hexokinase isoenzyme 1
YLR258W	*GSY2*	2.4	3.1		2.0	2.2		1.6		carbohydrate metabolism	Glycogen synthase; expression induced by glucose limitation
YOR120W	*GCY1*	2.2	3.5		2.0	1.9		1.4	•	carbohydrate metabolism	Glycerol dehydrogenase
YPR001W	*CIT3*	2.2	2.0		1.9	2.3		3.1		carbohydrate metabolism	Dual specificity mitochondrial citrate and methylcitrate synthase
YIL099W	*SGA1*	2.2	2.2		2.8	3.0		2.2		carbohydrate metabolism	Intracellular sporulation-specific glucoamylase
YGR087C	*PDC6*	2.1	1.9		3.7	2.9		2.9		carbohydrate metabolism	Minor isoform of pyruvate decarboxylase
YER062C	*GPP2*	2.1	2.4	•	1.7	2.4		2.0		carbohydrate metabolism	DL-glycerol-3-P phosphatase involved in glycerol biosynthesis
YDL022W	*GPD1*	2.0	2.2	•	2.2	2.9		2.0		carbohydrate metabolism	NAD-dependent glycerol-3-phosphate dehydrogenase
YKL096W	*CWP1*	3.5	3.8	•	2.4	1.4	•	4.1		cell wall	Cell wall mannoprotein that localizes to birth scars
YLR194C	---	2.6	6.3	•	2.0	1.6	•	2.1		cell wall	Structural constituent of the cell wall
YLR414C	*PUN1*	2.3	4.0	•	4.1	3.7		2.1		cell wall	Plasma membrane protein with a role in cell wall integrity
YER150W	*SPI1*	2.3	6.5		2.7	4.2		2.4		cell wall	GPI-anchored cell wall protein involved in weak acid resistance
YGR032W	*GSC2*	2.2	5.2	•	2.9	2.9		2.4		cell wall	Catalytic subunit of 1,3-beta-glucan synthase
YAL061W	*BDH2*	5.6	7.8		5.8	7.7		2.9	•	generation of energy	Putative medium-chain alcohol dehydrogenase
YDL085W	*NDE2*	3.7	4.1		3.4	3.0		2.8		generation of energy	Mitochondrial external NADH dehydrogenase
YOR374W	*ALD4*	3.3	3.7	•	3.7	3.6		4.0		generation of energy	Mitochondrial aldehyde dehydrogenase
YML054C	*CYB2*	3.0	5.1		4.1	4.0		2.5		generation of energy	Cytochrome b2 (L-lactate cytochrome-c oxidoreductase)
YPL171C	*OYE3*	2.8	3.2		2.9	3.3		2.0		generation of energy	Conserved NADPH oxidoreductase
YDL130W-A	*STF1*	2.4	2.4		1.7	2.1		2.2		generation of energy	Protein involved in regulation of the mitochondrial F1F0-ATP synthase
YPR151C	*SUE1*	2.4	2.2		2.1	2.1		3.1		generation of energy	Protein required for degradation of unstable forms of cytochrome c

YDL222C	*FMP45*	13.9	21.6		9.5	17.1	6.5	•	lipid metabolism	Protein required for sporulation and maintaining sphingolipid content
YNL194C	---	11.1	24.4		12.9	21.8	3.6	•	lipid metabolism	Protein required for sporulation and maintaining sphingolipid content
YGL205W	*POX1*	4.6	7.5		8.3	6.8	12.5		lipid metabolism	Fatty-acyl coenzyme A oxidase
YIL160C	*POT1*	2.8	4.1		3.5	2.5	4.1		lipid metabolism	3-ketoacyl-CoA thiolase with broad chain length specificity
YNL202W	*SPS19*	2.3	2.6		2.0	2.4	3.0		lipid metabolism	Peroxisomal 2,4-dienoyl-CoA reductase
YHR160C	*PEX18*	2.2	2.1		3.1	2.8	3.6		lipid metabolism	Peroxin; required for targeting of peroxisomal matrix proteins
YDL223C	*HBT1*	3.8	7.1		3.1	4.0	2.0	•	mating	Shmoo tip protein, substrate of Hub1p ubiquitin-like protein
YBR230C	*OM14*	2.0	2.3		1.8	2.0	1.6		mitochondrial	Mitochondrial outer membrane receptor for cytosolic ribosomes
YDL204W	*RTN2*	4.8	7.0		3.1	4.0	1.5	•	organelle organization	Protein involved in maintenance of tubular ER morphology
YBL078C	*ATG8*	3.0	3.2	•	2.5	2.8	2.5		organelle organization	Component of autophagosomes and Cvt vesicles
YHR138C	---	2.1	2.9		2.9	2.7	2.3		organelle organization	Protein of unknown function
YLR178C	*TFS1*	2.8	5.2		3.1	3.1	2.0		protein modification	Inhibitor of carboxypeptidase Y (Prc1p), and Ras GAP (Ira2p)
YEL060C	*PRB1*	2.1	2.6	•	2.3	2.3	1.9		protein modification	Vacuolar proteinase B with H3 N-terminal endopeptidase activity
YLL039C	*UBI4*	2.0	3.1		1.8	1.8	3.5		protein modification	Ubiquitin
YDL024C	*DIA3*	3.9	4.0		4.9	6.3	2.3	•	pseudohyphal growth	Protein of unknown function, involved in pseudohyphal growth
YMR169C	*ALD3*	8.9	21.2	•	9.2	12.6	1.8	•	response to stress	Cytoplasmic aldehyde dehydrogenase
YPL223C	*GRE1*	6.4	11.8		8.0	9.1	3.9	•	response to stress	Hydrophilin essential in desiccation-rehydration process
YGR088W	*CTT1*	5.4	8.5	•	5.5	8.4	1.2	•	response to stress	Cytosolic catalase T
YDR453C	*TSA2*	4.5	7.2		4.4	5.4	1.9	•	response to stress	Stress inducible cytoplasmic thioredoxin peroxidase
YGR213C	*RTA1*	4.0	10.9		8.6	10.3	3.3		response to stress	Protein involved in 7-aminocholesterol resistance
YMR174C	*PAI3*	4.0	3.9		3.0	3.0	2.2	•	response to stress	Cytoplasmic proteinase A (Pep4p) inhibitor
YIL101C	*XBP1*	3.2	3.5		3.2	3.5	2.8		response to stress	Transcriptional repressor that binds promoter of cyclin genes
YBR072W	*HSP26*	3.2	6.3		3.0	4.3	1.7	•	response to stress	Small heat shock protein (sHSP) with chaperone activity
YMR095C	*SNO1*	3.0	4.8		5.3	3.9	2.9		response to stress	Protein involved in pyridoxine metabolism
YGR144W	*THI4*	2.9	3.3		2.5	3.0	3.2		response to stress	Protein involved in the formation of the thiazole moiety
YDL110C	*TMA17*	2.4	2.2		2.0	2.3	2.6		response to stress	ATPase chaperone that adapts proteasome assembly to stress
YMR096W	*SNZ1*	2.4	4.8		3.0	2.4	2.4		response to stress	Protein involved in vitamin B6 biosynthesis
YDL243C	*AAD4*	2.3	2.5		3.0	3.4	2.4		response to stress	Putative dehydrogenase involved in oxidative stress response
YDR074W	*TPS2*	2.3	2.3		2.5	2.7	1.8		response to stress	Phosphatase subunit of the trehalose-6-P synthase complex
YMR040W	*YET2*	2.2	2.4		3.3	2.9	2.8		response to stress	Protein of unknown function that may interact with ribosomes
YMR322C	*SNO4*	2.1	1.9		2.2	2.4	2.5		response to stress	Possible chaperone and cysteine protease
YML100W	*TSL1*	2.1	2.1	•	2.2	2.7	1.8		response to stress	Large subunit of trehalose 6-phosphate synthase complex
YGR008C	*STF2*	2.1	2.4		2.4	2.7	2.4		response to stress	Protein involved in resistance to desiccation stress
YER037W	*PHM8*	2.0	2.3		2.2	3.6	2.9		response to stress	Lysophosphatidic acid (LPA) phosphatase
YDR001C	*NTH1*	2.0	2.4		1.7	1.6	2.4		response to stress	Neutral trehalase, degrades trehalose
YHR087W	*RTC3*	7.4	10.8	•	6.6	10.0	10.3		RNA metabolism	Protein of unknown function involved in RNA metabolism
YOR173W	*DCS2*	3.2	4.6	•	3.1	3.4	2.1		RNA metabolism	m(7)GpppX pyrophosphatase regulator

YPL123C	*RNY1*	2.1	2.7		1.6	2.3	2.0		RNA metabolism	Vacuolar RNase of the T(2) family
YGR043C	*NQM1*	10.6	22.1	•	12.2	16.3	7.4		signal transduction	Transaldolase of unknown function
YOR134W	*BAG7*	8.1	24.2		6.8	7.6	5.5		signal transduction	Rho GTPase activating protein (RhoGAP)
YGL121C	*GPG1*	3.8	4.3		2.8	4.0	4.3		signal transduction	Proposed gamma subunit of the heterotrimeric G protein
YDL214C	*PRR2*	2.7	2.8		2.9	4.0	2.2		signal transduction	Protein kinase that inhibits pheromone induced signalling
YHR139C	*SPS100*	14.6	27.4		10.5	12.6	6.7	•	sporulation	Protein required for spore wall maturation
YOL047C	*LDS2*	2.4	1.9		2.1	1.9	2.1		sporulation	Protein Involved in spore wall assembly
YOR177C	*MPC54*	2.2	2.0		2.1	2.1	2.1		sporulation	Component of the meiotic outer plaque
YGR059W	*SPR3*	2.0	1.4		2.5	2.5	1.9		sporulation	Sporulation-specific homolog of the CDC3/10/11/12 family of genes
YIR017C	*MET28*	2.9	1.8		3.3	3.9	3.4		transcription	bZIP transcriptional activator in the Cbf1p-Met4p-Met28p complex
YJL089W	*SIP4*	2.3	1.0		2.4	2.7	3.5		transcription	C6 zinc cluster transcriptional activator
YHR096C	*HXT5*	38.2	71.0		23.0	40.6	12.7	•	transport	Hexose transporter with moderate affinity for glucose
YNR002C	*ATO2*	7.5	11.3		13.0	14.9	9.6		transport	Putative transmembrane protein involved in export of ammonia
YDR536W	*STL1*	6.6	5.6		8.4	10.9	4.0	•	transport	Glycerol proton symporter of the plasma membrane
YNL093W	*YPT53*	4.9	11.1		10.4	11.8	3.0	•	transport	Stress-induced Rab family GTPase
YOR348C	*PUT4*	3.8	3.8		4.7	5.6	2.7		transport	Protein required for high-affinity transport of proline
YGR243W	*MPC3*	3.8	4.6		3.1	4.1	5.6		transport	Highly conserved subunit of mitochondrial pyruvate carrier
YPR194C	*OPT2*	2.9	2.4		2.2	3.3	1.5	•	transport	Oligopeptide transporter
YBR294W	*SUL1*	2.9	3.0		6.5	4.5	7.8		transport	High affinity sulfate permease of the SulP anion transporter family
YCR010C	*ADY2*	2.6	3.3		2.7	2.7	4.3		transport	Acetate transporter required for normal sporulation
YKL217W	*JEN1*	2.6	2.7		3.6	3.5	3.1		transport	Monocarboxylate/proton symporter of the plasma membrane
YGR121C	*MEP1*	2.4	2.7		5.1	6.2	2.1		transport	Ammonium permease
YOR273C	*TPO4*	2.3	1.8		2.3	2.7	2.9		transport	olyamine transporter of the major facilitator superfamily
YKR039W	*GAP1*	2.3	1.9		2.7	2.7	2.1		transport	General amino acid permease
YOR100C	*CRC1*	2.3	1.1		2.2	2.0	3.3		transport	Mitochondrial inner membrane carnitine transporter
YLR417W	*VPS36*	2.0	1.9		2.6	2.3	2.1		transport	Component of the ESCRT-II complex
YMR107W	*SPG4*	18.6	46.5		18.0	22.5	15.5		unknown	Protein required for survival at high temperature during stationary phase
YDR034W-B	---	14.3	48.9		16.1	31.7	12.3		unknown	Predicted tail-anchored plasma membrane protein
YOL084W	*PHM7*	8.4	14.2		9.2	13.6	3.1	•	unknown	Protein of unknown function
YGR052W	*FMP48*	5.7	6.8	•	8.5	15.2	4.1		unknown	Putative protein of unknown function
YML128C	*MSC1*	5.6	11.7	•	6.1	8.0	3.0	•	unknown	Protein of unknown function
YBR285W	---	4.7	6.2		8.1	6.1	4.1		unknown	Putative protein of unknown function
YPL054W	*LEE1*	4.5	3.9		5.4	8.2	3.6		unknown	Zinc-finger protein of unknown function
YLR327C	*TMA10*	4.0	7.1	•	8.8	9.9	8.6		unknown	Protein of unknown function that associates with ribosomes
YIL057C	*RGI2*	3.9	2.6		4.2	3.3	8.3		unknown	Protein of unknown function
YLR267W	*BOP2*	3.8	6.3		4.8	6.1	4.6		unknown	Protein of unknown function
YNL195C	---	3.3	3.8		3.6	4.0	1.3	•	unknown	Protein of unknown function

YNR034W-A	---	3.3	4.6		2.5	4.0	1.8	•	unknown	Putative protein of unknown function
YGR066C	---	3.3	3.0		4.0	3.3	5.0		unknown	Putative protein of unknown function
YKL107W	---	3.2	4.2		3.6	2.9	4.9		unknown	Putative short-chain dehydrogenase/reductase
YHR140W	---	3.2	5.9		3.7	4.0	2.4		unknown	Putative integral membrane protein of unknown function
YER067W	*RGI1*	3.1	4.5		3.1	3.8	1.6	•	unknown	Protein of unknown function
YGR174W-A	---	3.1	2.2		2.3	2.8	3.5		unknown	Putative protein of unknown function
YLR149C	---	2.9	4.1		3.1	4.0	3.5		unknown	Protein of unknown function
YBR085C-A	---	2.9	3.4		3.2	3.7	2.9		unknown	Protein of unknown function
YEL073C	---	2.8	3.7		3.1	3.3	3.1		unknown	Putative protein of unknown function
YDR018C	---	2.8	2.6		1.9	2.3	1.8		unknown	Probable membrane protein
YLR031W	---	2.7	3.8		2.8	3.0	3.4		unknown	Putative protein of unknown function;
YHL021C	*AIM17*	2.7	4.3		4.2	4.2	2.0		unknown	Putative protein of unknown function
YOR161C	*PNS1*	2.6	2.9	•	2.3	2.3	1.6	•	unknown	Protein of unknown function
YMR196W	---	2.5	4.0		2.6	2.7	2.2		unknown	Putative protein of unknown function
YLR312C	---	2.5	2.9		1.9	2.7	3.4		unknown	Putative protein of unknown function
YMR090W	---	2.5	3.3	•	2.4	2.4	1.6		unknown	Putative protein of unknown function
YMR118C	*SHH3*	2.5	1.8		1.9	1.6	3.8		unknown	Putative mitochondrial inner membrane protein
YEL057C	---	2.4	2.5		2.3	2.7	2.4		unknown	Protein of unknown function involved in telomere maintenance
YML131W	---	2.4	2.5	•	1.8	2.3	2.5		unknown	Protein of unknown function
YOR289W	---	2.4	2.8		2.4	2.8	2.7		unknown	Putative protein of unknown function
YHR097C	---	2.3	3.2	•	3.0	3.2	1.8		unknown	Putative protein of unknown function
YKR046C	*PET10*	2.3	5.7		1.6	2.3	2.3		unknown	Protein of unknown function that localizes to lipid particles
YBR076W	*ECM8*	2.2	1.9		2.2	1.9	2.4		unknown	Non-essential protein of unknown function
YBR056W-A	---	2.2	5.6		4.7	4.9	1.5	•	unknown	Protein of unknown function
YJR008W	*MHO1*	2.2	3.1		2.1	1.9	2.5		unknown	Protein of unknown function
YJL163C	---	2.1	1.9		2.0	2.3	1.9		unknown	Putative protein of unknown function
YDL169C	*UGX2*	2.0	2.6		1.9	2.3	2.2		unknown	Protein of unknown function
YPL272C	*PBI1*	2.0	5.6		2.7	3.9	2.3		unknown	Putative protein of unknown function
YPR159C-A	---	2.0	1.2		2.4	2.1	1.7		unknown	Protein of unknown function

Open reading frames the transcripts of which were induced at least twofold in the WT BY4741 strain after caspofungin treatment (15 ng/ml 2h) and their corresponding expression ratios (+/- drug) in the *slt2* Δ, *rlm1* Δ, and *msn2/4* Δ mutant strains under the same conditions are shown. The column labeled WT AMC includes gene expression data from microarray experiments corresponding to wild-type cells grown in the presence or absence of aminocandin (15 ng/ml 2h). The column labeled CR-ZY includes, labeled with a black dot, the genes that were expressed in response to Congo red (CR) or/and zymolyase (ZY) treatment, as previously described[1,2]. Functional groups and description were assigned based on the information provided by the Saccharomyces Genome Database (SGD). Genes were grouped together on the basis of their dependence on activation by CAS on the Slt2 MAPK (Slt2-dependent genes highlighted in gray). Those genes dependent on Rlm1 or Msn2/4 for activation by CAS are labeled with a black dot.

Table S2

ORF	Gene	Ratio AMC	Ratio CAS	Functional group	Description
YNR001C	*CIT1*	2.01	1.80	Acetyl-coA metabolism	Citrate synthase
YAL054C	*ACS1*	2.01	1.80	Acetyl-coA metabolism	Acetyl-coA synthetase isoformo
YBR117C	*TKL2*	10.38	**6.32**	amino acid metabolism	Transketolase
YDR380W	*ARO10*	6.30	**5.57**	amino acid metabolism	Phenylpyruvate decarboxylase
YGL184C	*STR3*	3.00	**2.68**	amino acid metabolism	Peroxisomal cystathionine beta-lyase
YHR137W	*ARO9*	2.28	**2.62**	amino acid metabolism	Aromatic aminotransferase II
YJL088W	*ARG3*	4.98	**2.68**	amino acid metabolism	Ornithine carbamoyltransferase
YJR078W	*BNA2*	3.17	**2.54**	amino acid metabolism	Tryptophan 2,3-dioxygenase or indoleamine 2,3-dioxygenase
YKR076W	*ECM4*	2.96	**2.30**	amino acid metabolism	Omega class glutathione transferase
YLR142W	*PUT1*	10.74	**6.61**	amino acid metabolism	Protein involved in utilization of proline as sole nitrogen source
YMR250W	*GAD1*	3.45	**2.56**	amino acid metabolism	Glutamate decarboxylase
YJR010W	*MET3*	2.05	1.88	amino acid metabolism	Catalyzes the primary step of intracellular sulfate activation
YFL030W	*AGX1*	2.13	1.71	amino acid metabolism	Catalyzes the synthesis of glycine from glyoxylate
YER081W	*SER3*	2.60	1.60	amino acid metabolism	Catalyzes the first step in serine and glycine biosynthesis
YKL218C	*SRY1*	2.29	1.27	amino acid metabolism	Deaminates L-threo-3-hydroxyaspartate to form oxaloacetate and ammonia
YOL058W	*ARG1*	2.38	0.93	amino acid metabolism	Arginosuccinate synthetase
YDL022W	*GPD1*	2.18	**2.03**	carbohydrate metabolism	NAD-dependent glycerol-3-phosphate dehydrogenase
YEL011W	*GLC3*	2.75	**2.58**	carbohydrate metabolism	Glycogen branching enzyme, involved in glycogen accumulation
YER062C	*GPP2*	2.36	**2.06**	carbohydrate metabolism	DL-glycerol-3-P phosphatase involved in glycerol biosynthesis
YFR053C	*HXK1*	3.42	**2.58**	carbohydrate metabolism	Hexokinase isoenzyme 1
YGL156W	*AMS1*	4.22	**2.98**	carbohydrate metabolism	Nannosidase involved in free oligosaccharide (fOS) degradation
YGR248W	*SOL4*	4.76	**4.10**	carbohydrate metabolism	6-phosphogluconolactonase
YGR256W	*GND2*	6.42	**5.46**	carbohydrate metabolism	6-phosphogluconate dehydrogenase
YIL099W	*SGA1*	2.17	**2.18**	carbohydrate metabolism	Intracellular sporulation-specific glucoamylase
YIL107C	*PFK26*	2.21	**2.02**	carbohydrate metabolism	6-phosphofructo-2-kinase
YJL153C	*INO1*	41.30	**13.27**	carbohydrate metabolism	Inositol 1-phosphate synthase
YJL155C	*FBP26*	2.80	**2.45**	carbohydrate metabolism	Fructose-2,6-bisphosphatase
YLR258W	*GSY2*	3.09	**2.35**	carbohydrate metabolism	Glycogen synthase; expression induced by glucose limitation
YMR105C	*PGM2*	4.29	**2.80**	carbohydrate metabolism	Catalyzes the conversion from glucose-1-P to glucose-6-P
YOR120W	*GCY1*	3.52	**2.21**	carbohydrate metabolism	Glycerol dehydrogenase
YPL088W	---	11.31	**5.66**	carbohydrate metabolism	Putative aryl alcohol dehydrogenase
YPR001W	*CIT3*	2.03	**2.20**	carbohydrate metabolism	Dual specificity mitochondrial citrate and methylcitrate synthase
YPR160W	*GPH1*	9.44	**6.85**	carbohydrate metabolism	Glycogen phosphorylase required for the mobilization of glycogen
YER054C	*GIP2*	2.13		carbohydrate metabolism	Putative regulatory subunit of the protein phosphatase Glc7p
YFR015C	*GSY1*	2.12	1.93	carbohydrate metabolism	Glycogen synthase with similarity to Gsy2p

YPR184W	*GDB1*	2.56	1.92	carbohydrate metabolism	Glycogen debranching enzyme required for glycogen degradation
YBR006W	*UGA2*	2.28	1.91	carbohydrate metabolism	Protein involved in the utilization of gamma-aminobutyrate (GABA) as a nitrogen source
YIR038C	*GTT1*	2.40	1.82	carbohydrate metabolism	ER associated glutathione S-transferase capable of homodimerization
YDL021W	*GPM2*	2.36	1.81	carbohydrate metabolism	Homolog of Gpm1p phosphoglycerate mutase
YFR017C	*IGD1*	2.16	1.80	carbohydrate metabolism	Cytoplasmic protein that inhibits Gdb1p glycogen debranching activity
YJL052W	*TDH1*	2.71	1.74	carbohydrate metabolism	Glyceraldehyde-3-phosphate dehydrogenase involved in glycolysis and gluconeogenesis
YNL274C	*GOR1*	2.03	1.70	carbohydrate metabolism	Glyoxylate reductase
YGL062W	*PYC1*	2.07	1.50	carbohydrate metabolism	Cytoplasmic enzyme that converts pyruvate to oxaloacetate
YGL157W	*ARI1*	2.13	1.70	carbohydrate metabolism	Member of the short-chain dehydrogenase/reductase superfamily
YDR055W	*PST1*	5.80	**2.93**	cell wall	Cell wall protein that contains a putative GPI-attachment site
YDR077W	*SED1*	2.31	**2.03**	cell wall	Major stress-induced structural GPI-cell wall glycoprotein
YER150W	*SPI1*	6.47	**2.33**	cell wall	GPI-anchored cell wall protein involved in weak acid resistance
YGR032W	*GSC2*	5.21	**2.24**	cell wall	Catalytic subunit of 1,3-beta-glucan synthase
YGR189C	*CRH1*	2.66	**2.06**	cell wall	Chitin transglycosylase; transfer of chitin to beta(1-6) and (1-3) glucans
YIR039C	*YPS6*	3.33	**3.15**	cell wall	Putative GPI-anchored aspartic protease
YJL160C	*PIR5*	2.94	**2.55**	cell wall	Member of the PIR family of cell wall proteins
YKL096W	*CWP1*	3.79	**3.50**	cell wall	Cell wall mannoprotein that localizes to birth scars
YKL104C	*GFA1*	2.51	**2.40**	cell wall	Glutamine-fructose-6-P amidotransferase of chitin biosynthesis
YKL163W	*PIR3*	47.27	**18.79**	cell wall	O-glycosylated covalently-bound cell wall protein
YKR061W	*KTR2*	3.96	**2.96**	cell wall	Mannosyltransferase involved in N-linked protein glycosylation
YKR091W	*SRL3*	10.41	**7.27**	cell wall	GTB motif (G1/S transcription factor binding) containing protein
YLR121C	*YPS3*	4.19	**2.88**	cell wall	Member of family of proteases involved in cell wall maintenance
YLR194C	---	6.25	**2.57**	cell wall	Structural constituent of the cell wall
YLR414C	*PUN1*	3.96	**2.34**	cell wall	Plasma membrane protein with a role in cell wall integrity
YNL192W	*CHS1*	2.81	**2.14**	cell wall	Chitin synthase I
YDR261C	*EXG2*	2.08	1.89	cell wall	Exo-1,3-beta-glucanase involved in cell wall beta-glucan assembly
YEL058W	*PCM1*	2.36	1.88	cell wall	N-acetylglucosamine-phosphate mutase involved in the biosynthesis of chitin
YNL160W	*YGP1*	2.52	1.71	cell wall	Cell wall-related secretory glycoprotein
YBR005W	*RCR1*	2.24	1.24	cell wall	Protein of the ER membrane involved in cell wall chitin deposition
YAL061W	*BDH2*	7.78	**5.57**	generation of energy	Putative medium-chain alcohol dehydrogenase
YDL085W	*NDE2*	4.12	**3.69**	generation of energy	Mitochondrial external NADH dehydrogenase
YDL130W-A	*STF1*	2.44	**2.39**	generation of energy	Protein involved in regulation of the mitochondrial F1F0-ATP synthase
YDL181W	*INH1*	2.95	**2.26**	generation of energy	Protein that inhibits ATP hydrolysis by the F1F0-ATP synthase
YML054C	*CYB2*	5.06	**3.01**	generation of energy	Cytochrome b2 (L-lactate cytochrome-c oxidoreductase)
YOR374W	*ALD4*	3.74	**3.29**	generation of energy	Mitochondrial aldehyde dehydrogenase
YPL171C	*OYE3*	3.16	**2.79**	generation of energy	Conserved NADPH oxidoreductase
YPR151C	*SUE1*	2.18	**2.39**	generation of energy	Protein required for degradation of unstable forms of cytochrome c

YKL151C	---	2.60		generation of energy	Putative protein of unknown function
YNL009W	*IDP3*	2.12	1.97	generation of energy	Peroxisomal NADP-dependent isocitrate dehydrogenase
YMR315W	---	2.27	1.91	generation of energy	Protein with NADP(H) oxidoreductase activity
YDL222C	*FMP45*	21.57	**13.92**	lipid metabolism	Protein required for sporulation and maintaining sphingolipid content
YGL205W	*POX1*	7.51	**4.63**	lipid metabolism	Fatty-acyl coenzyme A oxidase
YHR160C	*PEX18*	2.13	**2.18**	lipid metabolism	Peroxin; required for targeting of peroxisomal matrix proteins
YHR209W	*CRG1*	16.05	**9.50**	lipid metabolism	S-AdoMet-dependent methyltransferase involved in lipid homeostasis
YIL160C	*POT1*	4.14	**2.75**	lipid metabolism	3-ketoacyl-CoA thiolase with broad chain length specificity
YNL194C	---	24.43	**11.08**	lipid metabolism	Protein required for sporulation and maintaining sphingolipid content
YNL202W	*SPS19*	2.63	**2.26**	lipid metabolism	Peroxisomal 2,4-dienoyl-CoA reductase
YPL110C	*GDE1*	2.17	**2.21**	lipid metabolism	Glycerophosphocholine (GroPCho) phosphodiesterase
YER015W	*FAA2*	2.64	1.81	lipid metabolism	Long chain fatty acyl-CoA synthetase
YMR008C	*PLB1*	2.28	1.63	lipid metabolism	Phospholipase B (lysophospholipase) involved in lipid metabolism
YPL057C	*SUR1*	2.13	1.54	lipid metabolism	Mannosylinositol phosphorylceramide (MIPC) synthase catalytic subunit
YKR053C	*YSR3*	2.13	1.27	lipid metabolism	Phosphatase involved in sphingolipid metabolism
YDL223C	*HBT1*	7.15	**3.83**	mating	Shmoo tip protein, substrate of Hub1p ubiquitin-like protein
YIL117C	*PRM5*	6.74	**4.26**	mating	Pheromone-regulated protein
YJL108C	*PRM10*	6.72	**5.97**	mating	Pheromone-regulated protein
YBR230C	*OM14*	2.28	**2.03**	mitochondrial	Mitochondrial outer membrane receptor for cytosolic ribosomes
YIL136W	*OM45*	2.29	1.98	mitochondrial	Protein of unknown function
YGR110W	*CLD1*	2.36	1.74	mitochondrial	Mitochondrial cardiolipin-specific phospholipase
YPR002W	*PDH1*	2.54	1.58	mitochondrial	Mitochondrial protein that participates in respiration
YBR214W	*SDS24*	2.08	1.87	morphogenesis	Homologs of the Schizosaccharomyces pombe Sds23 protein
YMR124W	*EPO1*	2.09	1.58	morphogenesis	Protein involved in septin-ER tethering
YBL078C	*ATG8*	3.24	**2.98**	organelle organization	Component of autophagosomes and Cvt vesicles
YDL204W	*RTN2*	7.05	**4.76**	organelle organization	Protein involved in maintenance of tubular ER morphology
YHR138C	---	2.90	**2.10**	organelle organization	Protein of unknown function
YOL083W	*ATG34*	2.10	1.80	organelle organization	Receptor protein involved in selective autophagy during starvation
YOR152C	*ATG40*	2.14	1.53	organelle organization	Autophagy receptor with a role in endoplasmic reticulum degradation
YEL060C	*PRB1*	2.61	**2.09**	protein modification	Vacuolar proteinase B with H3 N-terminal endopeptidase activity
YLL039C	*UBI4*	3.06	**2.04**	protein modification	Ubiquitin
YLR178C	*TFS1*	5.17	**2.79**	protein modification	Inhibitor of carboxypeptidase Y (Prc1p), and Ras GAP (Ira2p)
YPL052W	*OAZ1*	2.32	**2.00**	protein modification	Regulator of ornithine decarboxylase Spe1
YBR280C	*SAF1*	2.29	1.87	protein modification	F-Box protein involved in proteasome-dependent degradation of Aah1p
YPR030W	*CSR2*	2.66	1.70	protein modification	Nuclear ubiquitin protein ligase binding protein
YBR169C	*SSE2*	2.07	1.56	protein modification	Member of the heat shock protein 70 family that may be involved in protein folding
YER103W	*SSA4*	2.07	1.38	protein modification	Plays a role in SRP-dependent cotranslational protein-membrane targeting

YOR302W	---	2.08	1.53	protein synthesis	Regulates translation of the CPA1 mRNA
YDL024C	*DIA3*	3.99	**3.86**	pseudohyphal growth	Protein of unknown function, involved in pseudohyphal growth
YBR072W	*HSP26*	6.30	**3.15**	response to stress	Small heat shock protein (sHSP) with chaperone activity
YBR203W	*COS111*	2.68	**2.20**	response to stress	Protein required for antifungal drug ciclopirox olamine resistance
YCR104W	*PAU3*	2.23	**2.32**	response to stress	Member of the seripauperin multigene family
YDL110C	*TMA17*	2.18	**2.45**	response to stress	ATPase chaperone that adapts proteasome assembly to stress
YDL243C	*AAD4*	2.47	**2.34**	response to stress	Putative dehydrogenase involved in oxidative stress response
YDR001C	*NTH1*	2.35	**2.01**	response to stress	Neutral trehalase, degrades trehalose
YDR074W	*TPS2*	2.30	**2.33**	response to stress	Phosphatase subunit of the trehalose-6-P synthase complex
YDR453C	*TSA2*	7.21	**4.54**	response to stress	Stress inducible cytoplasmic thioredoxin peroxidase
YDR533C	*HSP31*	2.18	**2.18**	response to stress	Methylglyoxalase that converts methylglyoxal to D-lactate
YER037W	*PHM8*	2.25	**2.04**	response to stress	Lysophosphatidic acid (LPA) phosphatase
YFL014W	*HSP12*	25.86	**13.26**	response to stress	Protein involved in maintaining membrane organization
YGR008C	*STF2*	2.37	**2.10**	response to stress	Protein involved in resistance to desiccation stress
YGR088W	*CTT1*	8.46	**5.45**	response to stress	Cytosolic catalase T
YGR144W	*THI4*	3.31	**2.91**	response to stress	Protein involved in the formation of the thiazole moiety
YGR213C	*RTA1*	10.95	**4.03**	response to stress	Protein involved in 7-aminocholesterol resistance
YIL101C	*XBP1*	3.49	**3.16**	response to stress	Transcriptional repressor that binds promoter of cyclin genes
YML100W	*TSL1*	2.11	**2.11**	response to stress	Large subunit of trehalose 6-phosphate synthase complex
YMR040W	*YET2*	2.44	**2.22**	response to stress	Protein of unknown function that may interact with ribosomes
YMR095C	*SNO1*	4.77	**3.03**	response to stress	Protein involved in pyridoxine metabolism
YMR096W	*SNZ1*	4.84	**2.41**	response to stress	Protein involved in vitamin B6 biosynthesis
YMR169C	*ALD3*	21.16	**8.88**	response to stress	Cytoplasmic aldehyde dehydrogenase
YMR174C	*PAI3*	3.93	**3.97**	response to stress	Cytoplasmic proteinase A (Pep4p) inhibitor
YMR175W	*SIP18*	8.97	**10.47**	response to stress	Phospholipid-binding hydrophilin
YOL052C-A	*DDR2*	9.43	**6.99**	response to stress	Multi-stress response protein
YPL223C	*GRE1*	11.77	**6.39**	response to stress	Hydrophilin essential in desiccation-rehydration process
YPR005C	*HAL1*	3.81	**2.46**	response to stress	Cytoplasmic protein involved in halotolerance
YBL064C	*PRX1*	2.24	1.97	response to stress	Mitochondrial peroxiredoxin (1-Cys Prx) with thioredoxin peroxidase activity
YPL230W	*USV1*	2.07	1.95	response to stress	Putative transcription factor containing a C2H2 zinc finger
YNR064C	---	3.47	1.93	response to stress	Member of the alpha/beta hydrolase fold family
YOL151W	*GRE2*	2.42	1.92	response to stress	NADPH-dependent methylglyoxal reductase
YLR251W	*SYM1*	2.59	1.86	response to stress	Protein required for ethanol metabolism
YKL026C	*GPX1*	2.25	1.84	response to stress	Phospholipid hydroperoxide glutathione peroxidase induced by glucose starvation
YNL036W	*NCE103*	2.04	1.66	response to stress	Metalloenzyme that catalyzes CO2 hydration to bicarbonate
YML058W-A	*HUG1*	2.02	1.52	response to stress	Protein involved in the Mec1p-mediated checkpoint pathway that responds to DNA damage
YKL093W	*MBR1*	2.31	1.51	response to stress	Protein involved in mitochondrial functions and stress response

YFL056C	*AAD6*	2.01	1.39	response to stress	Putative aryl-alcohol dehydrogenase
YBR054W	*YRO2*	2.50	1.22	response to stress	Protein with a putative role in response to acid stress
YHR087W	*RTC3*	10.84	**7.38**	RNA metabolism	Protein of unknown function involved in RNA metabolism
YOR173W	*DCS2*	4.59	**3.18**	RNA metabolism	m(7)GpppX pyrophosphatase regulator
YPL123C	*RNY1*	2.74	**2.08**	RNA metabolism	Vacuolar RNase of the T(2) family
YML118W	*NGL3*	2.46	1.90	RNA metabolism	3'-5' exonuclease specific for poly-A RNAs
YCR073C	*SSK22*	2.97	**2.32**	signal transduction	MAP kinase kinase kinase of the HOG1 pathway
YDL214C	*PRR2*	2.77	**2.67**	signal transduction	Protein kinase that inhibits pheromone induced signalling
YDR085C	*AFR1*	9.76	**5.63**	signal transduction	Protein required for pheromone-induced projection formation
YGL121C	*GPG1*	4.28	**3.84**	signal transduction	Proposed gamma subunit of the heterotrimeric G protein
YGL248W	*PDE1*	2.00	**2.04**	signal transduction	Low-affinity cyclic AMP phosphodiesterase
YGR023W	*MTL1*	2.70	**2.73**	signal transduction	Plasma membrane sensor involved in cell integrity signaling
YGR043C	*NQM1*	22.13	**10.57**	signal transduction	Transaldolase of unknown function
YHR030C	*SLT2*	3.46	**2.67**	signal transduction	Serine/threonine MAP kinase of cell wall integrity pathway
YKL161C	*KDX1*	41.39	**14.91**	signal transduction	Protein kinase of the cell wall integrity pathway
YMR104C	*YPK2*	4.64	**3.63**	signal transduction	Protein kinase similar to serine/threonine protein kinase Ypk1
YOR134W	*BAG7*	24.17	**8.07**	signal transduction	Rho GTPase activating protein (RhoGAP)
YOR208W	*PTP2*	3.17	**2.70**	signal transduction	Phosphotyrosine-specific phosphatase involved in osmosensing
YPL089C	*RLM1*	2.50	**2.22**	signal transduction	MADS-box transcription factor activated by the MAP-kinase Slt2p
YNL053W	*MSG5*	2.06	1.84	signal transduction	Protein phosphatase that regulates and is regulated by Slt2p
YGR070W	*ROM1*	2.03	1.71	signal transduction	Guanine nucleotide exchange factor (GEF) for Rho1p
YGR161C	*RTS3*	2.66	1.70	signal transduction	Putative component of the protein phosphatase type 2A complex
YBR050C	*REG2*	2.15	1.69	signal transduction	Regulatory subunit of the Glc7p type-1 protein phosphatase
YLR120C	*YPS1*	2.05	1.64	signal transduction	Aspartic protease
YOL016C	*CMK2*	2.32	1.30	signal transduction	Calmodulin-dependent protein kinase, may play a role in stress response
YHL022C	*SPO11*	2.18	**2.52**	sporulation	Meiosis-specific protein that initiates meiotic recombination
YHR139C	*SPS100*	27.43	**14.65**	sporulation	Protein required for spore wall maturation
YNL294C	*RIM21*	2.29	**2.22**	sporulation	pH sensor molecule, component of the RIM101 pathway
YOL048C	*RRT8*	2.04	1.64	sporulation	Protein involved in spore wall assembly
YDR277C	*MTH1*	3.19	**2.24**	transcription	Negative regulator of the glucose-sensing signal transduction pathway
YKL062W	*MSN4*	2.37	1.86	transcription	Transcriptional activator related to Msn2p
YDR216W	*ADR1*	2.05	1.77	transcription	Carbon source-responsive zinc-finger transcription factor
YMR280C	*CAT8*	2.74	1.76	transcription	Transcriptional activator of genes under non-fermentative growth conditions
YBR182C	*SMP1*	2.11	1.75	transcription	Putative transcription factor involved in regulating the response to osmotic stress
YGR097W	*ASK10*	2.42	1.74	transcription	Component of the RNA polymerase II holoenzyme
YDR259C	*YAP6*	2.07	1.47	transcription	Basic leucine zipper (bZIP) transcription factor
YMR136W	*GAT2*	2.03	1.43	transcription	Protein containing GATA family zinc finger motifs

YAL053W	*FLC2*	2.65	**2.41**	transport	Putative calcium channel
YBR294W	*SUL1*	2.99	**2.91**	transport	High affinity sulfate permease of the SulP anion transporter family
YCL069W	*VBA3*	2.09	**4.46**	transport	Permease of basic amino acids in the vacuolar membrane
YCR010C	*ADY2*	3.27	**2.65**	transport	Acetate transporter required for normal sporulation
YDR342C	*HXT6*	3.95	**2.90**	transport	High-affinity glucose transporter
YDR536W	*STL1*	5.61	**6.62**	transport	Glycerol proton symporter of the plasma membrane
YGR121C	*MEP1*	2.69	**2.38**	transport	Ammonium permease
YGR166W	*TRS65*	2.36	**2.08**	transport	Component of transport protein particle (TRAPP) complex II
YGR243W	*MPC3*	4.56	**3.77**	transport	Highly conserved subunit of mitochondrial pyruvate carrier
YHR092C	*HXT4*	3.57	**2.09**	transport	High-affinity glucose transporter
YHR096C	*HXT5*	71.02	**38.25**	transport	Hexose transporter with moderate affinity for glucose
YIL023C	*YKE4*	2.42	**3.04**	transport	Zinc transporter; localizes to the ER
YKL217W	*JEN1*	2.73	**2.57**	transport	Monocarboxylate/proton symporter of the plasma membrane
YNL015W	*PBI2*	2.38	**2.23**	transport	Cytosolic inhibitor of vacuolar proteinase B (PRB1)
YNL093W	*YPT53*	11.09	**4.90**	transport	Stress-induced Rab family GTPase
YNL293W	*MSB3*	2.13	**2.66**	transport	Rab GTPase-activating protein; regulates endocytosis
YNR002C	*ATO2*	11.30	**7.47**	transport	Putative transmembrane protein involved in export of ammonia
YNR065C	---	3.43	**2.86**	transport	Protein of unknown function
YNR066C	---	2.71	**2.16**	transport	Putative membrane-localized protein of unknown function
YOR306C	*MCH5*	2.74	**2.11**	transport	Plasma membrane riboflavin transporter
YOR348C	*PUT4*	3.77	**3.83**	transport	Protein required for high-affinity transport of proline
YPR194C	*OPT2*	2.40	**2.91**	transport	Oligopeptide transporter
YMR192W	*GYL1*	2.18	1.84	transport	Putative GTPase activating protein (GAP) that may have a role in polarized exocytosis
YOL122C	*SMF1*	2.02	1.79	transport	Ion transporter with a broad specificity for di-valent and tri-valent metals
YLR080W	*EMP46*	2.42	1.74	transport	Membrane component of endoplasmic reticulum-derived COPII-coated vesicles
YDL234C	*GYP7*	2.14	1.68	transport	GTPase-activating protein for yeast Rab family members
YBR147W	*RTC2*	2.39	1.62	transport	Putative vacuolar membrane transporter for cationic amino acids
YOR328W	*PDR10*	2.13	1.44	transport	ABC (ATP-binding cassette) membrane pump involved in the pleiotropic drug resistance
YGR138C	*TPO2*	3.93	1.00	transport	Polyamine transporter of the major facilitator superfamily
YBR056W-A	---	5.63	**2.24**	unknown	Protein of unknown function
YBR085C-A	---	3.36	**2.90**	unknown	Protein of unknown function
YBR285W	---	6.20	**4.68**	unknown	Putative protein of unknown function
YCL049C	---	2.15	**2.08**	unknown	Protein of unknown function
YCR099C	---	2.19	**2.25**	unknown	Protein of unknown function
YDL169C	*UGX2*	2.55	**2.03**	unknown	Protein of unknown function
YDR018C	---	2.61	**2.76**	unknown	Probable membrane protein
YDR034W-B	---	48.87	**14.25**	unknown	Predicted tail-anchored plasma membrane protein

YDR070C	*FMP16*	10.24	**7.02**	unknown	Protein of unknown function
YEL057C	---	2.49	**2.44**	unknown	Protein of unknown function involved in telomere maintenance
YEL073C	---	3.71	**2.79**	unknown	Putative protein of unknown function
YER067W	*RGI1*	4.54	**3.14**	unknown	Protein of unknown function
YGL258W-A	---	2.09	**2.07**	unknown	Putative protein of unknown function
YGR052W	*FMP48*	6.80	**5.67**	unknown	Putative protein of unknown function
YGR066C	---	2.99	**3.28**	unknown	Putative protein of unknown function
YGR149W	---	2.33	**2.05**	unknown	Putative protein of unknown function
YGR174W-A	---	2.19	**3.11**	unknown	Putative protein of unknown function
YHL021C	*AIM17*	4.35	**2.65**	unknown	Putative protein of unknown function
YHR097C	---	3.16	**2.28**	unknown	Putative protein of unknown function
YHR140W	---	5.89	**3.18**	unknown	Putative integral membrane protein of unknown function
YIL029C	---	2.05	**2.35**	unknown	Putative protein of unknown function
YIL057C	*RGI2*	2.63	**3.86**	unknown	Protein of unknown function
YIL108W	---	2.55	**2.13**	unknown	Putative metalloendopeptidase
YJL103C	*GSM1*	2.76	**2.17**	unknown	Putative zinc cluster protein of unknown function
YJL107C	---	6.09	**6.03**	unknown	Putative protein of unknown function
YJL161W	*FMP33*	4.89	**3.56**	unknown	Putative protein of unknown function
YJR008W	*MHO1*	3.13	**2.20**	unknown	Protein of unknown function
YKL107W	---	4.25	**3.20**	unknown	Putative short-chain dehydrogenase/reductase
YKR046C	*PET10*	5.69	**2.27**	unknown	Protein of unknown function that localizes to lipid particles
YLR031W	---	3.81	**2.71**	unknown	Putative protein of unknown function;
YLR149C	---	4.05	**2.94**	unknown	Protein of unknown function
YLR267W	*BOP2*	6.34	**3.83**	unknown	Protein of unknown function
YLR312C	---	2.91	**2.48**	unknown	Putative protein of unknown function
YLR327C	*TMA10*	7.15	**3.96**	unknown	Protein of unknown function that associates with ribosomes
YML128C	*MSC1*	11.71	**5.63**	unknown	Protein of unknown function
YML131W	---	2.50	**2.37**	unknown	Protein of unknown function
YMR090W	---	3.27	**2.47**	unknown	Putative protein of unknown function
YMR107W	*SPG4*	46.54	**18.63**	unknown	Protein required for survival at high temperature during stationary phase
YMR196W	---	4.01	**2.49**	unknown	Putative protein of unknown function
YNL058C	---	4.18	**2.89**	unknown	Putative protein of unknown function
YNL115C	---	2.01	**2.02**	unknown	Putative protein of unknown function
YNL195C	---	3.76	**3.33**	unknown	Protein of unknown function
YNR034W-A	---	4.58	**3.30**	unknown	Putative protein of unknown function
YOL084W	*PHM7*	14.16	**8.41**	unknown	Protein of unknown function
YOL159C	---	3.98	**3.46**	unknown	Soluble protein of unknown function

YOR161C	*PNS1*	2.90	**2.60**	unknown	Protein of unknown function
YOR289W	---	2.82	**2.37**	unknown	Putative protein of unknown function
YPL054W	*LEE1*	3.93	**4.48**	unknown	Zinc-finger protein of unknown function
YPL067C	---	2.77	**2.19**	unknown	Putative protein of unknown function
YPL272C	*PBI1*	5.62	**2.02**	unknown	Putative protein of unknown function
YPR078C	---	3.52	**3.46**	unknown	Putative protein of unknown function
YGR236C	*SPG1*	2.67	1.98	unknown	Protein required for high temperature survival during stationary phase
YNR014W	---	2.30	1.94	unknown	Putative protein of unknown function
YDR540C	*IRC4*	2.11	1.93	unknown	Putative protein of unknown function
YMR081C	*ISF1*	2.58	1.92	unknown	Serine-rich, hydrophilic protein with similarity to Mbr1p
YBR033W	*EDS1*	2.46	1.92	unknown	Protein of unknown function
YOR137C	*SIA1*	2.40	1.91	unknown	Protein may involved in activation of the Pma1p plasma membrane H+-ATPase
YMR194C-B	*CMC4*	2.47	1.89	unknown	Protein that localizes to the mitochondrial intermembrane space
YCR101C	---	2.17	1.88	unknown	Putative protein of unknown function
YKL133C	---	2.39	1.87	unknown	Putative protein of unknown function
YLR271W	*CMG1*	2.02	1.86	unknown	Putative protein of unknown function
YPL119C-A	---	2.06	1.85	unknown	Putative protein of unknown function
YJL132W	---	2.47	1.85	unknown	Putative protein of unknown function
YPL186C	*UIP4*	2.42	1.82	unknown	Protein of unknown function that interacts with Ulp1p
YNL092W	---	2.08	1.81	unknown	Putative S-adenosylmethionine-dependent methyltransferase
YMR251W-A	*HOR7*	2.75	1.81	unknown	Protein of unknown function
YBR056W	---	2.02	1.80	unknown	Putative protein of unknown function
YGR201C	---	2.86	1.79	unknown	Putative protein of unknown function
YPL222W	*FMP40*	2.16	1.78	unknown	Putative protein of unknown function
YCL012C	---	2.19	1.77	unknown	Putative protein of unknown function
YMR085W	---	2.98	1.74	unknown	Putative protein of unknown function
YLR054C	*OSW2*	4.91	1.72	unknown	Protein of unknown function may to be involved in the assembly of the spore wall
YHR112C	---	2.02	1.70	unknown	Putative protein of unknown function
YJL213W	---	2.16	1.70	unknown	Putative protein of unknown function
YOR019W	---	2.51	1.69	unknown	Putative protein of unknown function
YHR033W	---	2.64	1.68	unknown	Putative protein of unknown function
YLR346C	*CIS1*	3.10	1.67	unknown	Putative protein of unknown function found in mitochondria
YGL053W	*PRM8*	2.44	1.64	unknown	Pheromone-regulated protein
YMR084W	---	2.79	1.64	unknown	Putative protein of unknown function
YOR220W	*RCN2*	2.44	1.64	unknown	Protein of unknown function
YLL056C	---	2.02	1.61	unknown	Putative protein of unknown function
YDL246C	*SOR2*	2.06	1.54	unknown	Protein of unknown function

YKL071W	---	2.54	1.50	unknown	Putative protein of unknown function
YJL144W	---	2.67	1.47	unknown	Cytoplasmic hydrophilin of unknown function
YJL016W	---	2.09	1.47	unknown	Putative protein of unknown function
YMR316W	*DIA1*	4.07	1.44	unknown	Protein of unknown function
YBR071W	---	2.30	1.34	unknown	Protein of unknown function found in the cytoplasm and bud neck
YOR385W	---	2.09	1.03	unknown	Putative protein of unknown function

The table includes the complete data set from the microarray experiments (see text for details) of genes showing significant upregulation of expression (ratio +/- drug ≥ 2) in the presence of aminocandin (AMC) in the wild type strain. Microarrays expression data corresponding to caspofungin (CAS) treatment for the AMC upregulated genes is also included. The genes that were also induced by CAS are highlighted in bold. Functional groups and description were assigned based on the information contained in the Saccharomyces Genome Database (SGD). Blank boxes denote missing values.

Supplementary references

1. García, R. *et al.* The global transcriptional response to transient cell wall damage in *Saccharomyces cerevisiae* and its regulation by the cell integrity signaling pathway. *J.Biol.Chem* **279,** 15183-15195 (2004).

2. García, R., Rodríguez-Peña, J. M., Bermejo, C., Nombela, C. & Arroyo, J. The high osmotic response and cell wall integrity pathways cooperate to regulate transcriptional responses to zymolyase-induced cell wall stress in *Saccharomyces cerevisiae. J.Biol.Chem* **284,** 10901-10911 (2009).
